# Naringin Relieves Diabetic Cardiac Autonomic Neuropathy Mediated by P2Y_14_ Receptor in Superior Cervical Ganglion

**DOI:** 10.3389/fphar.2022.873090

**Published:** 2022-04-21

**Authors:** Gan Tang, Lingzhi Pi, Hongmin Guo, Zihui Hu, Congfa Zhou, Qixing Hu, Hao Peng, Zehao Xiao, Zhihua Zhang, Miaomiao Wang, Taotao Peng, Jiaqi Huang, Shangdong Liang, Guilin Li

**Affiliations:** ^1^ Queen Mary School, Medical School of Nanchang University, Nanchang, China; ^2^ School of Basic Medicine, Medical School of Nanchang University, Nanchang, China; ^3^ Department of Physiology, Medical School of Nanchang University, Nanchang, China; ^4^ Department of Anatomy, Medical School of Nanchang University, Nanchang, China

**Keywords:** diabetic cardiac autonomic neuropathy, satellite glial cells, superior cervical ganglia, P2Y14 receptor, naringin, ferroptosis

## Abstract

Diabetes mellitus (DM), an emerging chronic epidemic, contributes to mortality and morbidity around the world. Diabetic cardiac autonomic neuropathy (DCAN) is one of the most common complications associated with DM. Previous studies have shown that satellite glial cells (SGCs) in the superior cervical ganglia (SCG) play an indispensable role in DCAN progression. In addition, it has been shown that purinergic neurotransmitters, as well as metabotropic GPCRs, are involved in the pathophysiological process of DCAN. Furthermore, one traditional Chinese medicine, naringin may potently alleviate the effects of DCAN. Ferroptosis may be involved in DCAN progression. However, the role of naringin in DCAN as well as its detailed mechanism requires further investigation. In this research, we attempted to identify the effect and relevant mechanism of naringin in DCAN mitigation. We observed that compared with those of normal subjects, there were significantly elevated expression levels of P2Y_14_ and IL-1β in diabetic rats, both of which were remarkably diminished by treatment with either P2Y_14_ shRNA or naringin. In addition, abnormalities in blood pressure (BP), heart rate (HR), heart rate variability (HRV), sympathetic nerve discharge (SND), and cardiac structure in the diabetic model can also be partially returned to normal through the use of those treatments. Furthermore, a reduced expression of NRF2 and GPX4, as well as an elevated level of ROS, were detected in diabetic cases, which can also be improved with those treatments. Our results showed that naringin can effectively relieve DCAN mediated by the P2Y_14_ receptor of SGCs in the SCG. Moreover, the NRF2/GPX4 pathway involved in ferroptosis may become one of the principal mechanisms participating in DCAN progression, which can be modulated by P2Y_14_-targeted naringin and thus relieve DCAN. Hopefully, our research can supply one novel therapeutic target and provide a brilliant perspective for the treatment of DCAN.

## Introduction

Diabetes mellitus, a collection of metabolic diseases related to chronic inflammation, is characterized by hyperglycemia and markedly contributes to mortality and morbidity (American Diabetes Association, 2011; Li et al., 2017). Based on the different etiologies underlying the disease, diabetes is classified into four categories, including 1) type 1 diabetes, which results from an insulin-synthesis deficiency of beta cells out of autoimmune disorders; 2) type 2 diabetes, which is due to cellular insulin-resistance activities; 3) gestational diabetes, which indicates the presence of glucose intolerance during pregnancy; and 4) the fourth group, which involves other types of diabetes (Deshpande et al., 2008; American Diabetes Association, 2011). Among the distinctive types of diabetes, the vast majority contribute to long-term organic damage and potentially provoke other serious diseases, in which diabetic cardiac autonomic neuropathy (DCAN) is a common and quite devastating consequence (Vinik and Ziegler, 2007; American Diabetes Association, 2011).

Diabetic cardiac autonomic neuropathy (DCAN), is defined as an “impairment of cardiovascular autonomic control in patients with established diabetes after excluding other causes” (Spallone et al., 2011; Dimitropoulos et al., 2014; Agashe and Petak, 2018). As one of the most common diseases that is strongly related to diabetes, DCAN frequently manifests in one-fourth of patients with type 1 diabetes and one-third of patients with type 2 diabetes (Vinik and Ziegler, 2007). In addition to its high prevalence, there are multiple devastating complications of DCAN, such as silent myocardial ischemia (MI), all of which immensely influence the normal life of patients (Kahn et al., 1986; Vinik and Ziegler, 2007; Fedorowski et al., 2010; Balcıoğlu and Muderrisoglu, 2015; Agashe and Petak, 2018). Behind the clinical manifestations and complications, there is a complicated pathogenesis for DCAN. In the pathological case of DCAN, both the diabetes-induced diffused damage and the disorder of functional balance between cardiac sympathetic and parasympathetic nervous systems significantly evoke DCAN (Vinik et al., 2013). From previous studies, during DCAN progression, sophisticated interactions among glycemic regulation, blood pressure, and neuronal death contribute to the destruction of autonomic nerve fibers. As a result, the autonomic nerve fibers that innervate the cardiovascular system are damaged, consequently resulting in dysfunctional clinical manifestations (Vinik and Ziegler, 2007; Pop-Busui, 2010; Agashe and Petak, 2018). Within the complex interactions, the vast majority of hypotheses have suggested that hyperglycemia acts as the primary cause that triggers self-damaging reactions (Pop-Busui, 2010; Vinik et al., 2013; Dimitropoulos et al., 2014; Agashe and Petak, 2018). After the initiation of self-damaging reactions, all mitochondrion-derived oxidative stress, diabetes-associated DNA damage and increased specific inflammatory cytokines (such as interleukin 6, and C-reactive protein) contribute to microvascular abnormalities. Consequently, defects in neuronal blood supply promote impairments in peripheral nerves, eventually resulting in neural dysfunction and death (The Diabetes Control and Complications Trial Research Group, 1998; Ramasamy et al., 2005; Vinik and Ziegler, 2007; Pop-Busui, 2010; Vinik et al., 2013; Dimitropoulos et al., 2014; Balcıoğlu and Muderrisoglu, 2015; Agashe and Petak, 2018).

While monitoring the progression of DCAN, the superior cervical ganglion (SCG) should never be neglected. The superior cervical ganglion (SCG), as the largest ganglion that exists on both sides of the neck, communicates with the vagus nerve and glossopharyngeal nerve (Fazliogullari et al., 2016; Mitsuoka et al., 2017). Investigators have found that the SCG innervates the myocardium, by which it can participate in cardiac excitatory transmission and the regulation of cardiac physiological activities (Fazliogullari et al., 2016; Zou et al., 2018). Furthermore, it is well understood that SCG can integrate information from extrinsic and intrinsic stimulation, such as irritation derived from DCAN, indicating SCG plays an indispensable role in the progression of DCAN (Zou et al., 2018).

The complicated regulatory role of SCG is determined by sophisticated constituents (Hanani, 2010; Zou et al., 2018). SCG not only consists of neurons but is also sheathed by glial cells, especially satellite glial cells (SGCs) (Hanani, 2010; Zou et al., 2018). There are two principal functional significances for SGCs. On the one hand, SGCs harbor the ability to express immunological molecules such as interleukin-6, suggesting that SGCs are essential in inflammatory processes and are relevant to DCAN pathogenesis (Hanani, 2010). On the other hand, complex communications among SGCs and enclosed neurons are established by gap junctions. The specialized gap junctions render metabolites and ions transported smoothly between cells, achieving efficient intercellular connection. Therefore, SGCs in the SCG may play an important role in DCAN progression after the activation of SGCs by receptors.

One of the most significant receptor families expressed on the surface of SGCs are P2 receptors, which are classified into inotropic ligand-gated receptors (P2X receptors) and metabotropic G-protein coupled receptors (P2Y receptors) (Kobayashi et al., 2006; Villa et al., 2010). The P2Y_14_ receptor is a novel member of the metabolic receptor family (Kobayashi et al., 2006; Villa et al., 2010). The P2Y_14_ receptor is essentially expressed in glial cells (such as SGCs) and inflammatory cells (such as neutrophils). It can be activated by uridine diphosphates (UDPs) and UDP-sugars and responds accurately to extracellular nucleotides, exerting striking effects in two main aspects (Cho et al., 2014; Lin et al., 2019a). First, the P2Y_14_ receptor mediates the inflammatory responses to cellular damage and tissue injury (Cho et al., 2014). Second, the P2Y_14_ receptor is involved in inflammatory cytokine release, indirectly assisting immune reactions (Lin et al., 2019b). Therefore, the P2Y_14_ receptor, which participates in inflammatory responses and immune reactions, is a critical element in the pathogenesis of DCAN and may be one target for DCAN prognosis and treatment.

During the process of finding effective medicines to extend the survival of individuals with diabetes, naringin was discovered and widely studied. Naringin, a traditional Chinese herbal medicine, is an important flavonoid extracted from Citrus plants and harbors diverse functions, including anti-inflammation, antioxidant stress, and the amelioration of metabolic syndromes (Chen et al., 2016; Li et al., 2017). First, anti-inflammatory naringin can effectively diminish the secretion of diabetes-associated inflammatory molecules, such as interleukin-6 (IL-6) and TNF-α, both of which contribute to insulin resistance and hyperglycemia (Hotamisligil et al., 1993; Dandona et al., 2004; Krogh-Madsen et al., 2006; Alam et al., 2014; Chen et al., 2016). Furthermore, the antioxidant naringin not only has a strong potential to scavenge free radicals, such as diabetes-induced reactive oxygen species (ROS) (Guimarães et al., 2010; Chen et al., 2016) but also enhances the activity of antioxidant enzymes. Both can effectively alleviate free radical damage and ameliorate diabetes (Jeon et al., 2002; Ali and El Kader, 2004; Punithavathi et al., 2008). Moreover, naringin can mitigate mitochondrial dysfunction by regaining mitochondrial homeostasis (Chen et al., 2016).

Previous studies have shown that iron accumulation occurs in type 2 diabetes (Sha et al., 2021), and numerous reactive species are involved in DCAN, both of which are reminiscent of reactive lipid species (RLS) as well as ferroptosis. Ferroptosis is an iron-dependent cell death driven by the accumulation of lipid peroxide and is regarded as a one novel form of necrosis (Dodson et al., 2019). In the case of ferroptosis, free iron accumulation (resulting from an altered iron transport system or breakdown of iron-containing proteins) can initiate the overwhelming lipid peroxidation reaction, a process by which ROS and reactive nitrogen species (RNS) react with polyunsaturated fatty acids (PUFAs) in membranes to generate lipid peroxides (Dodson et al., 2019). Normally, there are several mechanisms to defend against the oxidative stress, including 1) the glutathione pathway as aided by glutathione peroxidase 4 (GPX4); 2) the NADPH-FSP1-CoQ10 ferroptosis surveillance pathway; and 3) the tetrahydrobiopterin (GCH1/BH4) pathway (Yang et al., 2014; Doll et al., 2019; Kraft et al., 2020). They are all precisely controlled, and various regulators are involved, such as nuclear factor erythroid 2-related Factor 2 (NRF2), by which intracellular homeostasis of reactive species is maintained and cells survive (Yang et al., 2014; Doll et al., 2019; Kraft et al., 2020). Nevertheless, when dysfunction occurs, homeostasis is destroyed, and ferroptosis is initiated, resulting in cell death.

On the one hand, the role of SGCs involved in DCAN has been tested, and the P2Y_14_ receptor is endowed with essential modulatory effects (Hanani, 2010). On the other hand, naringin harbors both anti-inflammation and antioxidant effects, and can function on specific receptors to suppress glial cell expression (Ahmed et al., 2019). However, not only does the detailed naringin effects on DCAN require more clarification, but also the sophisticated signaling pathways affected by naringin require further study.

In this experiment, we further studied the effects of naringin on DCAN as well as an associated mechanism of naringin for P2Y_14_ receptors in SGCs. Here, blood pressure (BP), heart rate (HR), and heart rate variability (HRV) were measured, sympathetic nerve discharge (SND) was recorded, and concentrations of ROS were detected, all of which helped us assess cardiac and nerve injury as well as ferroptosis-based DCAN. Additionally, Western blotting and triple-labeled immunofluorescence were used to evaluate the expression of P2Y_14_, IL-1β, NRF2, and GPX4. The experiment attampts to assist the vast majority of individuals suffering from the devastating DCAN by means of exploring a novel medicine target, the P2Y_14_ receptor, and taking advantage of a new therapy based on naringin effects, which are of great significance for further research on DCAN and the establishment of a basis for better treatment of DCAN.

## Materials and Methods

### Animals and Established Groups: Six Groups Were Set up

Adult male Sprague-Dawley (SD) rats (180–250 g) were provided by the Center of Laboratory Animal Science of Nanchang University and were used for the following experiments. The treatment of the animals was supervised by the Animal Care and Use Committees of Nanchang University Medical Schools. After adaptation to the laboratory environment for 1 week, the rats in the two groups were fed with distinctive food. Rats assigned to the control group (Ctrl group, n = 15) were given available water and common food composed of 53% carbohydrate, 23% protein, and 5% fat, while rats assigned to the type 2 diabetic model (n = 75) were fed with a high-sugar and high-fat diet (consisting of 66.5% basal diet, 20% sugar, 10% oil, 2.5% cholesterol, and 1% sodium cholate) for 4 weeks. Then, streptozotocin (STZ, 30 mg/kg) was intraperitoneally administrated to the rats in the type 2 diabetic model to facilitate the establishment of diabetic rats. Subsequently, 1 week after injection of STZ, blood glucose values for all the rats were assessed, and established diabetic rats whose fasting plasma glucose (FPG) was higher than 7.8 mM were identified and selected. The collected diabetic rats were further fed a high-fat and high-sugar diet, which lasted 2 weeks.

To examine the effect of P2Y_14_ short hairpin RNA (P2Y_14_ shRNA) and the effect of naringin, rats assigned to the type 2 diabetic model (n = 75) were randomly and evenly allocated into five groups: the type 2 diabetic group (DM, n = 15), type 2 diabetic rats treated with P2Y_14_ shRNA (DM + P2Y_14_ shRNA, n = 15), type 2 diabetic rats treated with scramble shRNA negative control group (DM + NC shRNA, n = 15), type 2 diabetic rats treated with naringin (DM + naringin, n = 15) and type 2 diabetic rats treated with a PBS solution negative control group (DM + PBS, n = 15). During the establishment of the DM + P2Y_14_ shRNA and DM + NC shRNA groups, both the P2Y_14_ and scramble shRNA were provided by Novobio Company of Shanghai, and the Entranster™—*in vivo* transfection reagents were obtained from Engreen Company of Beijing. A mixture composed of 10 μg P2Y_14_ shRNA and 20 μl transfection reagent was injected into each superior cervical ganglion (SCG) of rats in the DM + P2Y_14_ shRNA group, while the mixture composed of 10 μg scramble shRNA and 20 μl transfection reagent was injected into each SCG of rats in the DM + NC shRNA group. During the establishment of the DM + naringin and DM + PBS groups, naringin was provided by Zhibiaohuachun Biotechnical Company of Chengdu. In our research, 100 mg/kg of naringin solution (Alboghobeish et al., 2019) was administrated intraperitoneally into each rat in the DM + naringin group, while proper PBS solution was injected intraperitoneally into each rat in the DM + PBS group. Rats from the two groups received intraperitoneal administration every day for 2 weeks. Following that, intraperitoneal injection with 50 mg/kg sodium pentobarbital to anesthetize rats was conducted and SCGs of rats in six groups were collected.

### Measurement of BP, HRV, and SND

The rat’s heart rate and blood pressure, including systolic blood pressure (SBP), diastolic blood pressure (DBP), and mean blood pressure (MBP) were sensitively detected using the indirect tail-cuff method (Softron BP-98 A, Softron Co., Tokyo, Japan).

HRV was assessed on the strength of the recorded electrocardiogram (ECG). After anesthetization of the rat by intraperitoneal injection of pentobarbital sodium (50 mg/kg), the electrodes were fixed in the corresponding limbs, after which the 5-min ECG was recorded. Subsequently, the total power frequency (TP, 0–0.5 Hz), very low frequency (VLF, 0.003–0.04 Hz), low frequency (LF, 0.04–0.15 Hz), and high frequency (HF, 0.15–0.40 Hz) were determined and calculated from the rat’s 5-min ECG recording, the former of which suggests sympathetic activity and the latter of which represents parasympathetic activity.

The postganglionic cervical SND was further analyzed. After anesthetization of the rat by intraperitoneal injection of pentobarbital sodium (50 mg/kg), the identified left cervical sympathetic nerve (which was immersed in warm paraffin) was attached to silver electrodes that were connected to the RM6240 system to record SND while the reference electrode was attached to the skin. The settings for the SND patterns were as follows: a time constant of 0.001 s, recording sensitivity of 25–50 μV, power gain of 200 μV, scanning speed of 200 ms/div, and frequency filtering of 3 kHz. The postganglionic cervical SND was analyzed and quantified as μvolts × seconds (μV s).

### Western Blotting

The isolated SCGs were used to extract total protein by homogenizing SCG samples through mechanical disruption in prepared lysis buffer (50 mM Tris-Cl, pH 8.0, 150 mM NaCl, 0.1% sodium dodecyl sulfate (SDS), 0.02% sodium deoxycholate, 1% Nonidet P-40, 1% phosphatase inhibitors, 1% protease inhibitors, 1 μg/ml aprotinin and 100 μg/ml phenylmethylsulfonyl fluoride). Then, incubation followed on ice lasting 40 min, after which centrifugation of lysates was performed at 12,000 × g in a 4°C environment for 15 min. Subsequently, the supernatants were collected, followed by measurement of the total protein concentration using a bicinchoninic acid assay reagent kit. The sample buffer (250 mM Tris-Cl, 200 mM dithiothreitol, 0.5% bromophenol blue, 10% SDS, and 50% glycerol) was used to dilate the supernatants, and heating was conducted at 95°C for 5 min for denaturation of the protein. The supernatant sample was stored in a −20°C environment before usage.

At the beginning of Western blotting, the prepared supernatant samples containing an equal amount of protein among the six groups were loaded onto 10% SDS-polyacrylamide gels for electrophoresis, followed by the electrotransference of the protein of interest from the gel onto polyvinylidene fluoride (PVDF) membranes. The PVDF membrane carrying proteins was blocked with 5% nonfat dried milk in 25 mM Tris-buffered saline (pH 7.2) plus 0.05% Tween-20 (TBST) at room temperature for more than 2 h. Subsequently, the membranes were incubated with primary rabbit antibodies against P2Y_14_ (1:800, Abcam company, United Kingdom), GFAP (1:800, Abcam company, United Kingdom), IL-1β (1:400, Boster Biological Technology Company, China), NRF2 (1:500, Proteintech company, China), and GPX4 (1:800, Proteintech company, China) in 4°C environments overnight. The membranes were incubated with horseradish peroxidase-conjugated goat anti-rabbit IgG (1:2,000, Beijing Zhongshan Biotechnology Company) (as a secondary antibody) in a 4°C environment for more than 2 h. Proteins of interest were detected by a chemiluminescence gel imaging system (XRS + , Bio-Rad Company, United States).β-actin acting as an internal control was continuously assessed a protocol similar to that mentioned above. Eventually, the analysis of protein expression based on integrated optical density (IOD) was performed by means of Image Pro-Plus software.

### Double-Labeled Immunofluorescence

After collection of SCGs, SCGs were subjected to washing (in PBS solution) and fixation (in PFA lasting 24 h at 4°C), and then transferred to 20% sucrose in 4% PFA (overnight). Subsequently, freezing microtome was used to section tissues and store the slide at −20°C, prepared for a further experiment to examine the colocalization of proteins in SCG.

The prepared slides were used for immunofluorescence to examine the colocalization of P2Y_14_ proteins with GFAP, a biomarker of SGCs. First, the slides were washed with PBS solution and fixed with 0.4% PFA for 15 min, followed by washing and punching with 0.3% Triton X-100 at regular temperature for 10 min. Second, goat serum was used to incubate tissues for blocking for 1 h at 37°C. Third, the slides were incubated with rabbit anti-P2Y_14_ (1:150, ab184411; Abcam, Cambridge, UK) and mouse anti-GFAP (1:150, Cat# ab64613; Abcam, Cambridge, UK) at 4°C overnight. Fourth, the sections were subsequently incubated with FITC (fluorescein isothiocyanate)-labeled goat anti-mouse IgG (1:150, Beijing Zhongshan Biotech Co.,) as well as TRITC (tetraethyl rhodamine isothiocyanate)-labeled goat anti-rabbit IgG (1:150, Beijing Zhongshan Biotech Co.,) for more than 2 h at 37°C. Fifth, DAPI staining solution was used to stain the nucleus of the cells for 5 min. Sixth, an anti-fading solution was used and fluorescent images were captured under fluorescence microscope (Olympus DP72, Japan) (using the same exposure, contrast, light, and hue settings).

### Chemofluorometric Kits

SCG homogenates were prepared and the level of ROS in each group was detected by a chemofluorometric kit (Cat. No. E004-1-1, Nanjingjiancheng, China). We followed the protocol of the chemofluorometric kit and evaluated the difference in the concentration of ferroptosis factor (ROS) in each group. The final results were analyzed by Image-Pro Plus software.

### Hematoxylin-Eosin Staining

After collection of cardiac apex tissue, the paraffin section was prepared following stepwise procedures, including fixation (by formalin), dehydration (by alcohol solution in different concentrations), clearing (with xylene), embedding (into paraffin), and sectioning (by machine). Subsequently, xylene was used to remove paraffin, and an alcohol solution was used for hydration. Finally, the slide was stained with hematoxylin and eosin solutions, followed by observation to understand the pathological changes in the cardiac histological structure.

### Molecular Docking

Molecular docking computations were finished relying on AutoDock 4.2 (Trott and Olson, 2010; Sheng et al., 2018). We performed the docking procedure and then obtained the proper structure of the ligand, used for calculation. The crystal structure of the human P2Y_14_ receptor was acquired from the Protein Data Bank, and the naringin-bound P2Y_14_ receptor was analyzed using AutoDock Tools.

### Statistical Analysis

Raw data were analyzed using SPSS software (IBM Corp., Inc., Armonk, NY, United States) and GraphPad Prism software, and all the values were expressed as the mean ± standard error of the mean (SEM). Significant differences were assessed by one-way ANOVA followed by Tukey’s post hoc tests. A *p* value <0.05 indicated the existence of a significant difference.

## Results

### The Expression of the P2Y_14_ Receptor in the Superior Cervical Ganglia

**FIGURE 1:**
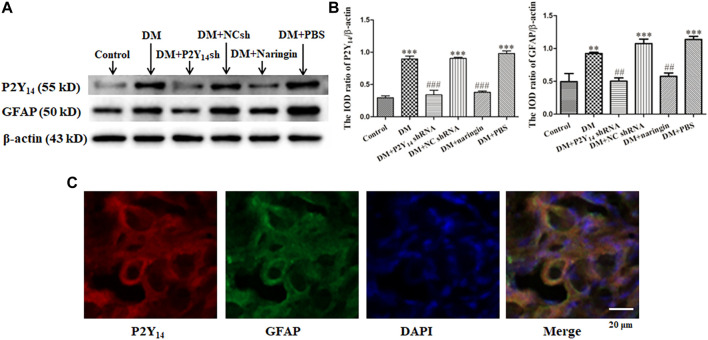
The expression levels of the P2Y_14_ receptor and GFAP in SCG. The protein expression levels of P2Y_14_ and GFAP were assessed by Western blotting **(A)**. The bar histogram shows the integrated optical density (IOD) ratio of P2Y_14_ and GFAP protein mass to β-actin protein mass (actin as an internal control for normalization) in each group **(B)**, and the values are the mean ± SEM from three independent experiments. ***p < 0.001 vs. Ctrl; ###*p* < 0.001 vs. DM. The expression of P2Y_14_, GFAP, and DAPI in the SCG was detected by immunofluorescence staining **(C)**. The red signal indicates P2Y_14_ staining with TRITC, the green signal represents GFAP staining with FITC, and the blue signal shows DAPI stain for labeling DNA. The merged image represents the three staining of P2Y_14_, GFAP, and DAPI. Scale bar, 20 μm.

Western blotting was used to analyze the protein expression levels of P2Y_14_ protein and GFAP in each group, and the integrated optical density (IOD) values were analyzed. The results showed that the expression levels of both P2Y_14_ protein and GFAP in the DM group were elevated compared with those in the control group (*p* < 0.01, Figures 1A,B). The expression levels of P2Y_14_ protein and GFAP in both the DM + P2Y_14_ shRNA group and DM + naringin group were significantly diminished in contrast to those in the DM group (*p* < 0.01). There were no significant differences observed among the DM, DM + NC shRNA, and DM + PBS groups (*p* > 0.05). There were no significant differences among the control, DM + P2Y_14_ shRNA, and DM + naringin groups (*p* > 0.05). All the results demonstrated that both treatment with P2Y_14_ shRNA and naringin may counteract the elevated expression of P2Y_14_ receptor protein and GFAP in the SCG of DM rats.

Double-labeled immunofluorescence was used to display the localization of P2Y_14_, GFAP and nuclei. Coexpression of P2Y_14_ and GFAP (one biomarker of SGCs) was observed in merged figures, indicating that P2Y_14_ was expressed by SGCs to participate in numerous SGC activities (Figure 1C).

### P2Y_14_ shRNA or Naringin Mitigated the Abnormal Changes in HR, SBP, DBP, and MBP

The cardiovascular parameters of HR, SBP, DBP and MBP in different groups are clearly shown in Table 1. The results revealed that all the HR, SBP, DBP, and MBP values in the DM group were overtly higher than those in the control group (*p* < 0.01), while the HR, SBP, DBP, and MBP values were significantly reduced in both the DM + P2Y_14_ shRNA group and the DM + naringin group compared with those of the DM group (*p* < 0.01). No significant difference in those cardiovascular parameters was discovered among the DM, DM + NC shRNA, and DM + PBS insertion: (*p* > 0.05). Furthermore, no significant differences were found among the control, DM + P2Y_14_ shRNA, and DM + naringin groups (*p* > 0.05). These results suggested increased excitability in the cardiac sympathetic nerve in DM rats, and the abnormality may be alleviated by targeting the P2Y_14_ receptor with either P2Y_14_ shRNA or naringin.

**TABLE 1:** Effects of P2Y_14_ shRNA and naringin on heart rate and blood pressure in rats.

Group	Heart rate (beat/min)	Blood pressure		
		SBP (mm Hg)	DBP (mm Hg)	MBP (mm Hg)
Control	322.70 ± 2.02	113.00 ± 1.81	87.10 ± 1.99	95.50 ± 1.87
DM	382.80 ± 2.43**	142.90 ± 1.88**	97.00 ± 1.29**	112.40 ± 1.42**
DM + P2Y_14_ shRNA	325.20 ± 1.95^##^	119.90 ± 1.33^##^	86.00 ± 1.39^##^	97.30 ± 1.10^##^
DM + NC shRNA	385.50 ± 2.51**	144.30 ± 2.00**	98.10 ± 2.08**	113.30 ± 2.01**
DM + naringin	325.60 ± 2.00^##^	117.20 ± 1.90^##^	87.90 ± 1.55^##^	97.70 ± 1.38^##^
DM + PBS	385.70 ± 2.15**	144.80 ± 1.63**	97.20 ± 1.69**	112.90 ± 1.65**

HR, heart rate; SBP, systolic blood pressure; DBP, diastolic blood pressure; MBP, mean blood pressure. Values are the mean ± SEM from ten observations in each group. **p < 0.01 vs. Ctrl; ##*p* < 0.01 vs. DM.

### P2Y_14_ shRNA or Naringin Mitigated the Abnormal Change in LF/HF Ratio of HRV

Table 2 represents the effect of both P2Y_14_ shRNA and naringin on the LF/HF ratio of HRV in each group. First, the values of TP, VLF, LF, and HF were all significantly decreased in the DM group compared with those in the control group (*p* < 0.01), and the abnormalities were mitigated in the DM + P2Y_14_ shRNA group as well as the DM + naringin group (*p* < 0.01). Moreover, compared with the control group, there was a remarkably elevated LF/HF ratio of HRV in the DM group (*p* < 0.01). After the treatment of DM rats with either P2Y_14_ shRNA or naringin, the abnormally increased LF/HF ratios of both groups were significantly diminished (*p* < 0.01). There were no significant differences observed among the DM, DM + NC shRNA, and DM + PBS groups (*p* > 0.05). There were no significant differences among the control, DM + P2Y_14_ shRNA, and DM + naringin groups (*p* > 0.05).

**TABLE 2:** Effects of P2Y_14_ shRNA on heart rate variability in rats.

	TP (ms^2^)	VLF (ms^2^)	LF (ms^2^)	HF (ms^2^)	LF/HF
Control	135.78 ± 1.71	125.94 ± 1.69	6.67 ± 0.17	12.93 ± 0.39	0.52 ± 0.02
DM	103.04 ± 2.56**	99.73 ± 1.91**	3.02 ± 0.17**	1.24 ± 0.08**	2.48 ± 0.13**
DM + P2Y_14_ shRNA	127.89 ± 1.81^##^	119.63 ± 1.87^##^	6.06 ± 0.13^##^	11.31 ± 0.41^##^	0.54 ± 0.01^##^
DM + NC shRNA	103.41 ± 2.30**	102.25 ± 2.33**	2.32 ± 0.11**	1.08 ± 0.05**	2.16 ± 0.10**
DM + naringin	132.11 ± 1.86^##^	123.84 ± 1.69^##^	6.35 ± 0.12^##^	12.03 ± 0.54^##^	0.54 ± 0.02^##^
DM + PBS	106.54 ± 3.01**	101.35 ± 2.28**	2.55 ± 0.11**	1.20 ± 0.08**	2.17 ± 0.09**

TP, total power frequency; VLF, very low frequency; LF, low frequency; HF, high frequency. Values are the mean ± SEM from ten observations in each group. **p < 0.01 vs. Ctrl; ##*p* < 0.01 vs. DM.

These results not only revealed that both sympathetic and parasympathetic nerves were injured and thus nerve activities were reduced in the case of DM, but also demonstrated relatively higher sympathetic tone and relatively lower parasympathetic tone in the DM group, because LF represented sympathetic activity and HF indicated parasympathetic activity, eventually giving rise to increased excitability in the cardiac sympathetic nerve in DM rats. Furthermore, the abnormalities of decreased TP, VLF, LF, and HF, as well as an increased LF/HF ratio, may be alleviated by targeting the P2Y_14_ receptor with either P2Y_14_ shRNA or naringin.

### P2Y_14_ shRNA or Naringin Mitigated the Abnormal Change in SND

**FIGURE 2:**
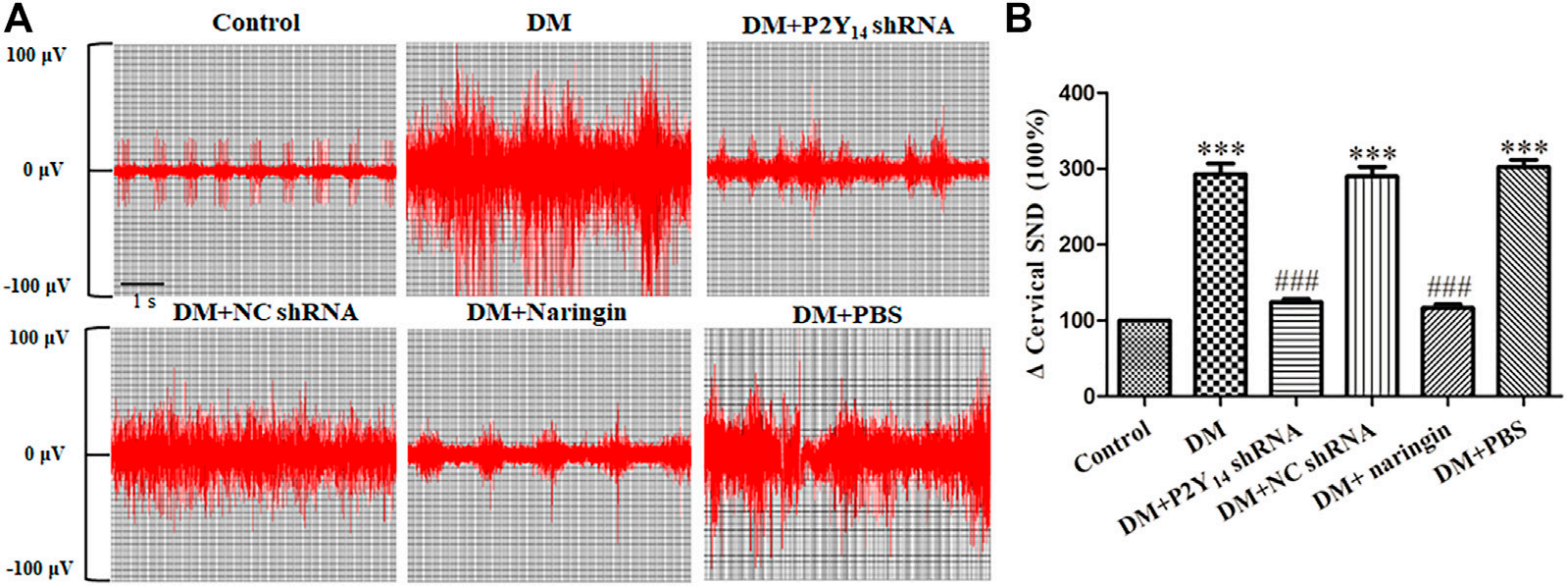
The effects of P2Y_14_ shRNA and naringin on cervical sympathetic nerve activity in type 2 diabetic rats. Representative images of postganglionic cervical SND of the SCG in each group **(A)**. Integrated cervical sympathetic discharge in each group **(B)**. The values are the means ± SEM (n = 10). ***p < 0.001 vs. Ctrl; ###*p* < 0.001 vs. DM.

Figure 2 represents the conditions of postganglionic cervical SND of the SCG in each group, and the effects of both P2Y_14_ shRNA and naringin in the corresponding groups are also shown in Figure 2. The results indicated that the postganglionic cervical SND in the DM group was evidently amplified in contrast to that in the control group (*p* < 0.001). Nevertheless, the abnormal cervical SND in DM rats treated with either P2Y_14_ shRNA or naringin was significantly diminished compared with untreated DM rats (*p* < 0.001). There were no significant differences observed among the DM, DM + NC shRNA, and DM + PBS groups or among the control, DM + P2Y_14_ shRNA, and DM + naringin groups (*p* > 0.05, Figure 2).

These results suggested increased excitability in the cardiac sympathetic nerve in DM rats, and the abnormality of it may be relieved by targeting the P2Y_14_ receptor with either P2Y_14_ shRNA or naringin.

### P2Y_14_ shRNA or Naringin Rescued the Pathological Cardiac Changes

**FIGURE 3:**
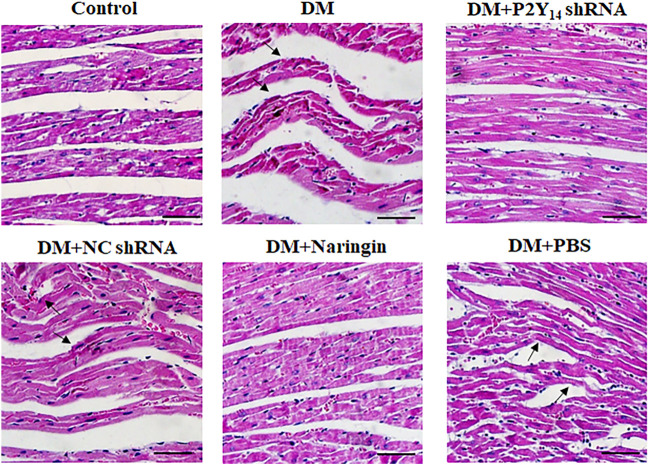
The cardiac tissue structure in each group after HE staining. The black arrows indicate overtly abnormal histological changes (destroyed myocardial fibers and distortion of fiber arrays) in the DM, DM + P2Y_14_ shRNA, and DM + PBS groups. Scale bar, 20 μm.

HE staining (Figure 3) was applied to assess the cardiac histological changes in the six groups, which are shown in Figure 3. Regular myocardial fibers and parallel arrays were observed in the control group. In contrast, myocardial fibers were destroyed and fiber arrays were distorted in the DM group. In the DM + P2Y_14_ shRNA group, the cardiac structure returned to normal overtly, which was similar to that of the DM + naringin group, indicating that treatment with either P2Y_14_ shRNA or naringin can effectively rescue the histological changes in cardiac structure caused by DCAN.

### P2Y_14_ shRNA or Naringin Diminished Expression of IL-1β in Superior Cervical Ganglia

**FIGURE 4:**
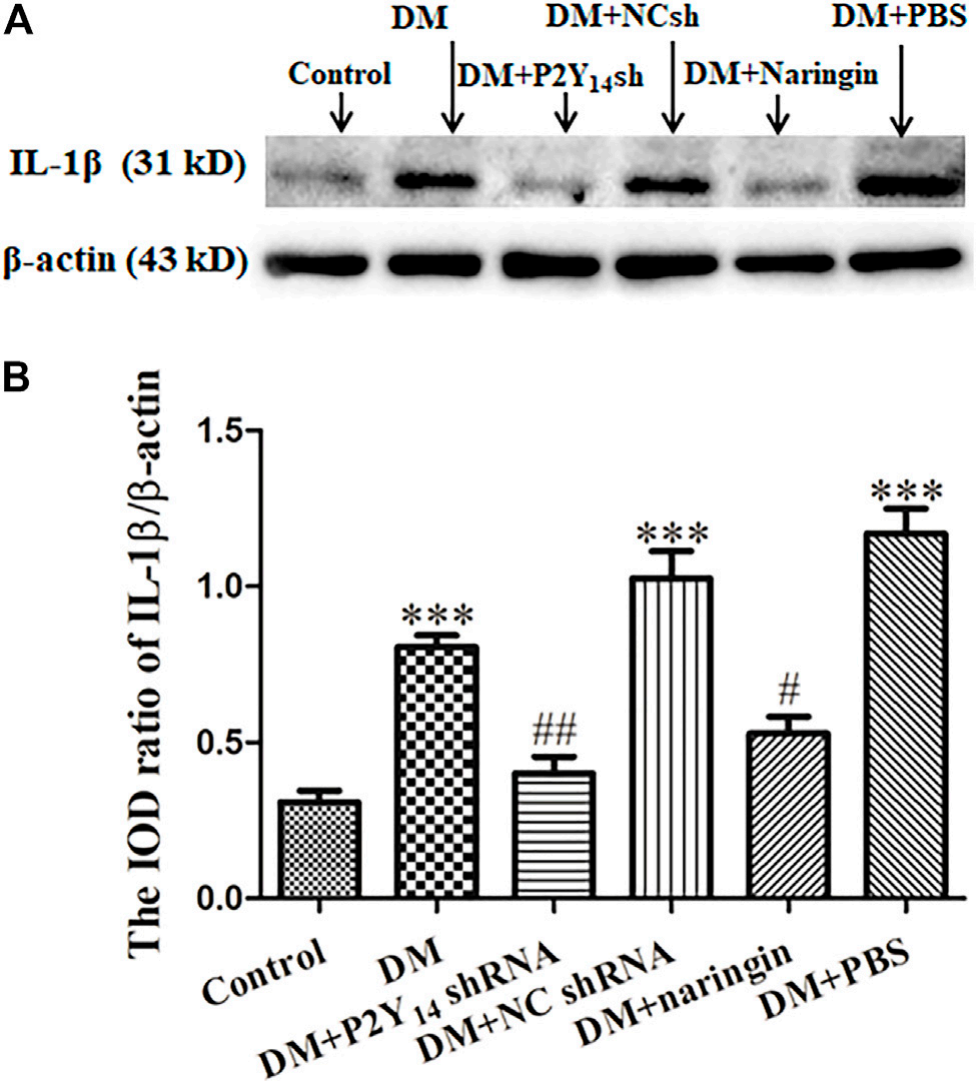
The effects of P2Y_14_ shRNA and naringin on the expression of IL-1β in the SCG of type 2 diabetic rats. The expression level of IL-1β protein was determined by Western blotting **(A)**. The bar histogram displays the IOD ratio of IL-1β protein mass to β-actin protein mass in each group **(B)**, and the values are the mean ± SEM from three independent experiments. ***p < 0.001 vs. Ctrl; #*p* < 0.05 vs. DM; ##*p* < 0.01 vs. DM.

Western blotting was used to analyze the expression levels of IL-1β protein in each group, and IOD values were analyzed. Figure 4A represents the images of IL-1β protein and β-actin protein from Western blotting, and Figure 4B shows the IOD ratio of IL-1β protein mass to β-actin protein mass which acts as an internal control for normalization. In contrast to the control group, the expression level of IL-1β protein was prominently increased in the DM group (*p* < 0.001). However, the expression levels of IL-1β protein in both the DM + P2Y_14_ shRNA and DM + naringin groups were significantly diminished compared with those of the DM group (*p* < 0.05). No significant differences were discovered among the control, DM + P2Y_14_ shRNA, and DM + naringin groups (*p* > 0.05, Figure 4).

It was shown that diabetes-induced activation of SCGs could produce various inflammatory factors, including IL-1β. Both treatment with P2Y_14_ shRNA and treatment with naringin may diminish the elevated expression of IL-1β in the SCG of DM rats.

### P2Y_14_ shRNA or Naringin Elevated Expression of NRF2 and GPX4 in Superior Cervical Ganglia

**FIGURE 5:**
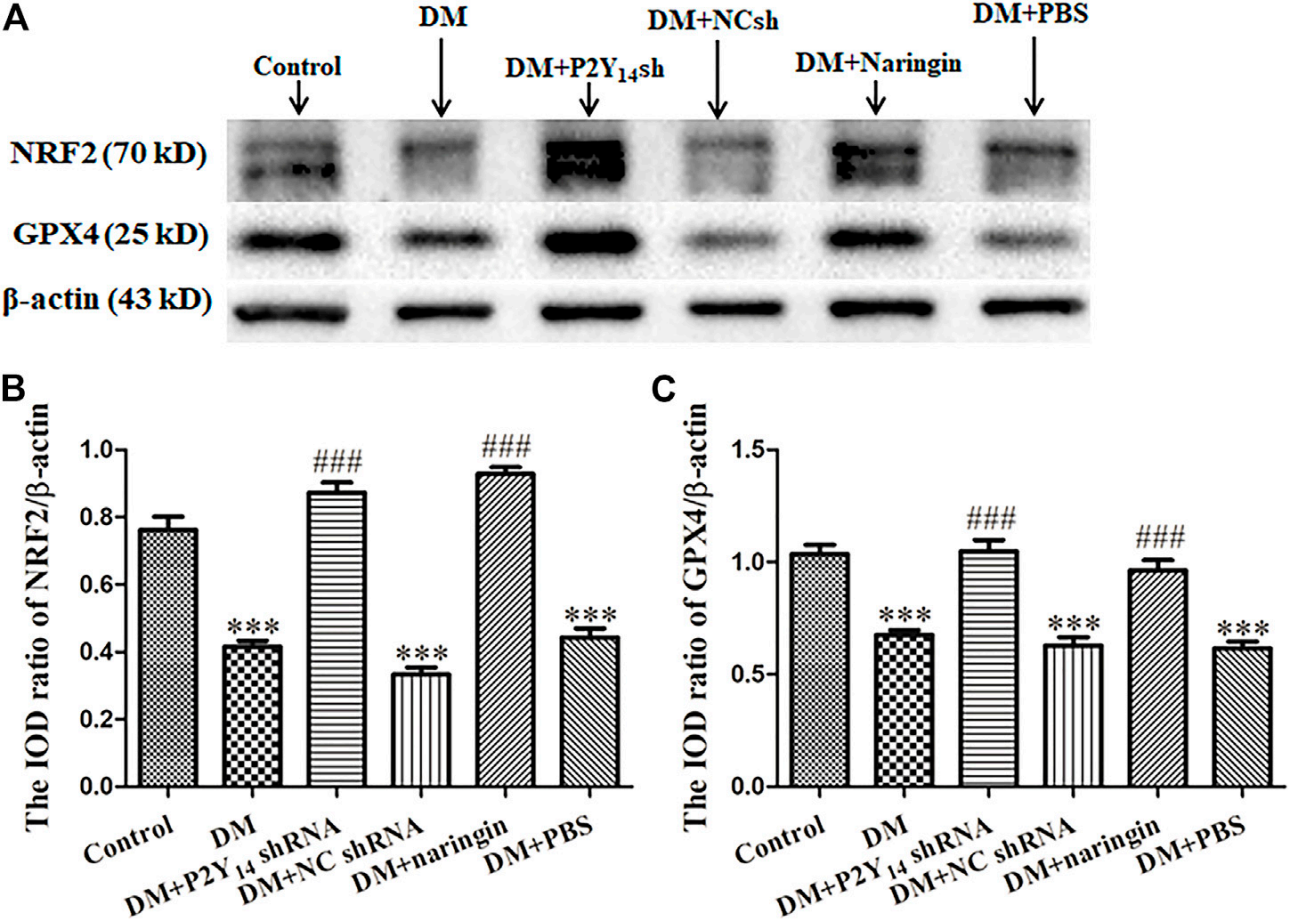
The effects of P2Y_14_ shRNA and naringin on the expression of NRF2 and GPX4 at the protein level in the SCG of type two diabetic rats. The protein expression levels of NRF2 and GPX4 were determined by Western blotting **(A)**. The bar histogram displays the IOD ratio of NRF2 **(B)** and GPX4 **(C)** and protein mass to β-actin protein mass in each group, and the values are the mean ± SEM from three independent experiments. ***p < 0.001 vs. Ctrl; ###*p* < 0.001 vs. DM.

Western blotting was applied to analyze the expression of NRF2 and GPX4 at the protein level in each group, and IOD values were analyzed. NRF2 or GPX4 protein expression was prominently diminished in the DM group compared to that of the control group (*p* < 0.001, Figure 5). Nevertheless, compared with the DM group, the expression levels of NRF2 and GPX4 protein in both the DM + P2Y_14_ shRNA and DM + naringin groups were significantly reversed (*p* < 0.001) thanks to the treatment. No significant differences were observed among the DM, DM + NC shRNA, and DM + PBS groups, and there were also no significant differences among the control, DM + P2Y_14_ shRNA and DM + naringin groups (*p* > 0.05, Figure 5).

The results showed that treatment with both P2Y_14_ shRNA and naringin may counteract the reduced expression of NRF2 and GPX4 at the protein level in the SCG of DM rats.

### P2Y_14_ shRNA or Naringin Eliminated ROS in Superior Cervical Ganglia

**FIGURE 6:**
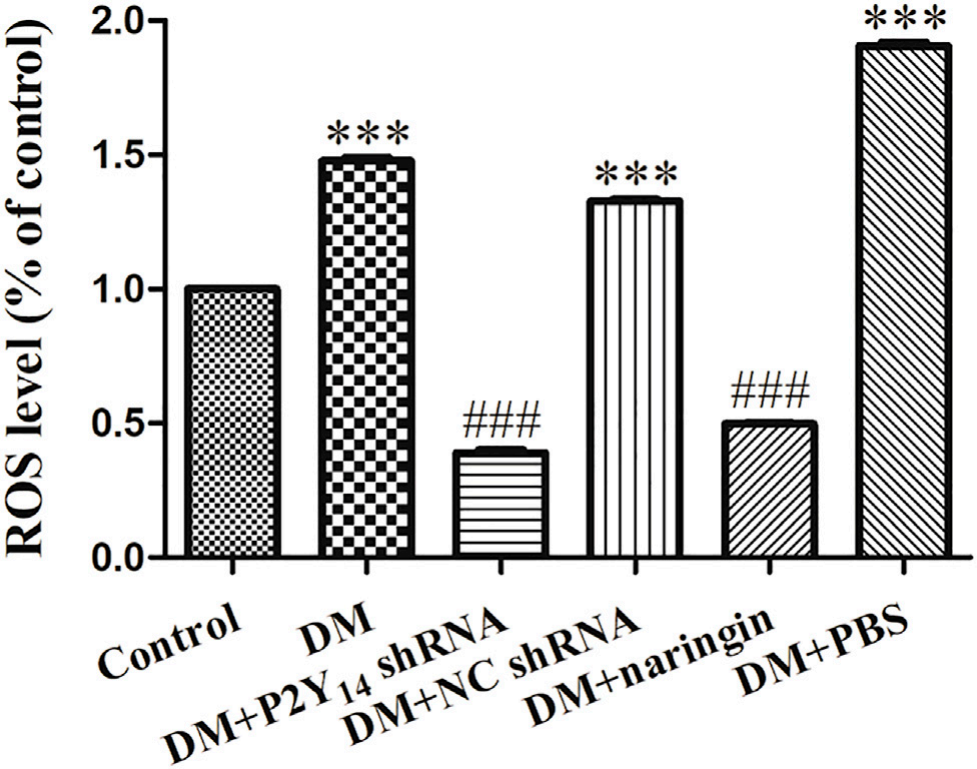
The relative ROS levels in each group compared with the control group. The values are the mean ± SEM from three independent experiments. ***p < 0.001 vs. Ctrl; ###*p* < 0.001 vs. DM.

A ROS chemofluorometric assay kit was used to compare the amount of ROS in the SCGs of each group. Figure 6 represents the relative amount of ROS in each group compared with that in the control group. Remarkably elevated amounts of ROS were observed in the DM group, DM + NC shRNA group, and DM + PBS group compared with the standardized ROS amount in the control group (*p* < 0.001), indicating that ROS-driven ferroptosis may be involved in DCAN. After treatment with either P2Y_14_ shRNA or naringin, the amount of ROS was reduced significantly (*p* < 0.001), suggesting that both treatments can eliminate ROS to some extent. There were no significant differences observed among the DM, DM + NC shRNA, and DM + PBS groups (*p* > 0.05, Figure 6).

### Molecular Docking of Naringin on P2Y_14_

**FIGURE 7:**
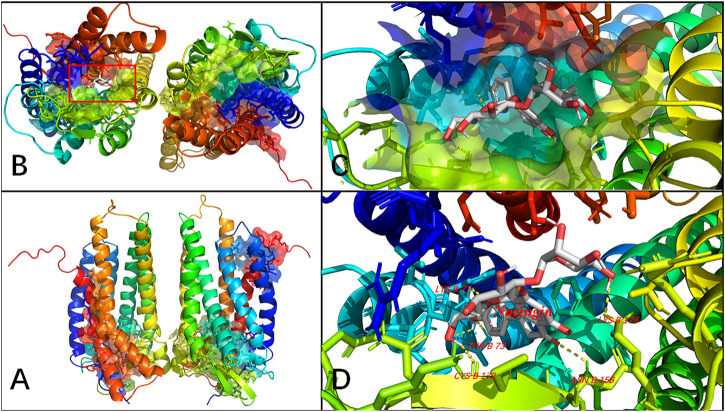
Molecular docking of naringin to the P2Y_14_ protein. The whole structure of the naringin-bound P2Y_14_ receptor **(A)**. The binding pocket is shown **(B)**, which was amplified in **(C)** and labeled in detail in **(D)**. The yellow dashed line indicates a possible hydrogen bond between the connected residues (B77 LYS, B73 THR, B176 LYS, B156 ASN, and B172 CYS) and the ligand. The data show that naringin can interact with the P2Y_14_ protein.

Molecular docking computations were applied to analyze the binding issue of naringin with the P2Y_14_ receptor. The results demonstrated that five amino acid residues may form hydrogen bonds with naringin (Figure 7), and the binding energy is -9.4 kcal/mol (Table 3), which reveals that strong chemical bonds exist between P2Y_14_ and naringin, indicating powerful interactions between the P2Y_14_ receptor and naringin.

**TABLE 3:** MOE score of the P2Y_14_ protein and naringin (kcal/mol).

Mode	Affinity (kcal/mol)	Dist from RMSD l.b	Best mode RMSD u.b
1	−9.4	0.000	0.000
2	−9.4	2.139	4.983
3	−8.8	1.585	4.703
4	−8.5	32.442	36.298
5	−8.3	31.725	36.355
6	−7.9	32.060	36.050
7	−7.8	29.070	34.847
8	−7.7	55.447	59.211
9	−7.7	28.053	33.501

The predicted binding affinity is in kcal/mol (energy). The RMSD values were calculated as related to the best mode and only used movable heavy atoms. Two variants of RMSD metrics are provided, RMSD l.b. (RMSD lower bound) and RMSD u.b. (RMSD upper bound), differing in how the atoms are matched in the distance calculation.

## Discussion

Within the pathological progression of DCAN, there are three prominent issues involved, namely 1) diabetes-driven SGC activation, 2) subsequent autonomic nerve damage (neuropathy), and 3) eventual cardiac damage. Naringin can effectively relieve DCAN by interacting with P2Y_14_ and then rescuing this pathological progression.

1) Diabetes-driven SGC activation via the P2Y_14_ receptor causes cardiac damage, which can be mitigated by P2Y_14_-targeted naringin.

Due to diabetes, abundant nucleotides are released from stressed cells, such as damaged neuronal soma in the nervous system, after which the exported nucleotides work as a “danger signal” to “talk” with other cells via purinergic receptor activation (Hochhauser et al., 2013; Lazarowski and Harden, 2015). Two categories of purinergic receptors can be activated, namely, ionotropic receptor (P2X receptor) and metabotropic GPCR (P2Y receptor), both of which are significant in the regulation of neuronal activities and interactions between neurons and glial cells (Calvert et al., 2004; Kobayashi et al., 2013; Rivera et al., 2016). Among the various purinergic receptors, the P2Y_14_ receptor is a novel metabotropic GPCR that can be activated by extracellular uridine diphosphates (UDPs) and UDP-sugar conjugates (Dobolyi et al., 2011; Curet and Watters, 2018). The P2Y_14_ receptor is broadly distributed in glial cells (such as SGCs, astrocytes, and microglia), where it plays significant roles in inflammatory and immunological processes (Barrett et al., 2013; Kinoshita et al., 2013; Lazarowski and Harden, 2015; Lin et al., 2019b). In our research, the localization of the P2Y_14_ receptor in the SCG was reidentified by double immunofluorescence labeling. The expression level of P2Y_14_ was consistent with that of GFAP, a biomarker of satellite glial cell (SGC) activation (Feldman-Goriachnik and Hanani, 2021), indicating that the P2Y_14_ receptor plays an essential role in the activation of SGCs. Compared with normal rats, the P2Y_14_ receptor was highly expressed and SGC cells were overtly activated in the case of diabetes. Furthermore, there was overtly elevated expressions of the P2Y_14_ receptor in diabetic rats compared with that of normal rats, and treatment with either P2Y_14_ shRNA or naringin effectively diminished the overexpression in diabetic rats, indicating that the naringin can effectively relieve DCAN by downregulating the expression of the P2Y_14_ receptor in the SCG.

After extracellular UDP and UDP-sugar binding to P2Y_14_ receptors, natively coupled with G_i/o_ heterotrimers, P2Y_14_ receptors are activated and trigger potent suppression of adenylyl cyclase G_i/o_ (Carter et al., 2009; Fricks et al., 2009; Kobayashi et al., 2013; Lin et al., 2019b). Subsequently, downstream signaling pathways, such as the MAPK pathway, are modulated, consequently influencing cardiovascular activities and possibly causing cardiac damage (Fricks et al., 2009; Hochhauser et al., 2013; Lazarowski and Harden, 2015; Lin et al., 2019b). In our experiments, the results of HR, BP (involving SBP, DBP, and MBP) and cardiac histological structure suggest the presence of cardiac abnormalities in the diabetic model, which were prominently mitigated after treatment with either P2Y_14_ shRNA or naringin. Therefore, it was suggested that naringin could clearly rescue cardiac damage in DCAN by targeting the P2Y_14_ receptor.

2) After SGC activation, autonomic nerve damage follows and plays a significant role in the progression of DCAN, which can be partially rescued by P2Y_14_-targeted naringin.

After the release of uridine nucleotides, the activation of the P2Y_14_ receptor brings about autonomic nerve damage and consequently causes neuropathy. In our research, spectral analysis of HRV acts as an effective way to understand the alterations of autonomic nerves. Spectral analysis of HRV not only showed that both sympathetic and parasympathetic nerve activities were impaired in subjects with diabetes but also confirmed that the balance between sympathetic and parasympathetic nerve activities is destroyed in diabetic conditions. However, the abnormally increased LF/HF ratio can be partially restored by treatment with either P2Y_14_ shRNA or naringin. Furthermore, SND also revealed abnormal nerve discharge activities in diabetic rats, which can return to normal in part after treatment with either P2Y_14_ shRNA or P2Y_14_-targeted naringin. Both of these results indicate that the P2Y_14_ receptor is involved in DCAN and that diabetes-induced nerve damage can be reversed by naringin-targeting P2Y_14_ receptors.

Within the whole process, satellite glial cells (SGCs) in the SCG can be activated and respond to environmental changes, consequently influencing nearby SGCs and neurons (Hanani, 2005; Hanani, 2010). First, activated SGCs induce the elevated expression of inflammatory cytokines, such as IL-1β, to mediate immunological responses and influence the excitability of surrounding neurons (Zhang et al., 2007; Ji et al., 2013; Ramesh et al., 2013; Lazarowski and Harden, 2015; Magni et al., 2015; Zhang et al., 2017; Wang et al., 2018; Lin et al., 2019b). Our research demonstrated that compared with that of the control group, the expression of IL-1β was evidently increased in activated SGCs (DM group), while there was a noteworthy reduction in the expression after either P2Y_14_ shRNA or naringin treatment. This suggests that overexpressed P2Y_14_ can play a role in DCAN by boosting the secretion of various inflammatory cytokines (such as IL-1β) in SGCs and that naringin can relieve the progression of DCAN by reducing their secretion from SGCs. Second, activated SGCs can also trigger the formation of gap junctions between SGCs as well as between SGCs and neurons, which can assist rapid transmission of chemical signals and thus directly modulate neuronal excitability (Warwick and Hanani, 2013; Lin et al., 2019b; Spray et al., 2019). Gap junctions among SGCs can join separated SGCs into functional networks, and gap junctions between SGCs and neurons can mediate neuron-glial bidirectional chemical signaling (Gu et al., 2010; Suadicani et al., 2010; Rozanski et al., 2012; Magni et al., 2015; Spray et al., 2019).

Both the release of inflammatory factors from SGCs and the formation of gap junctions between nerve cells give rise to autonomic nerve injury and neuropathy (Román-Pintos et al., 2016).

3) The NRF2/GPX4 pathway involved in ferroptosis may be one of the most prominent mechanisms involved in DCAN, and P2Y_14_-targeted naringin may alleviate DCAN by enhancing the NRF2/GPX4 pathway and suppressing the ferroptosis process.

There is abundant evidence suggesting that the ferroptosis-relevant NRF2/GPX4 pathway may play an indispensable role in nerve damage progression. In that case, the activities of antioxidant enzymes are overtly diminished, promoting the production of free radical (such as ROS) (Román-Pintos et al., 2016; de M Bandeira et al., 2013). Glutathione peroxidase 4 (GPX4), a potent antioxidant enzyme capable of removing lipid peroxides, may be involved in the pathological process (Liu and Wang, 2019). Furthermore, GPX4 can be directly or indirectly regulated by NRF2, a distinguished transcription factor that plays an important role in antioxidation. The activation of NRF2 can effectively protect against oxidative injuries in various pathological conditions, such as inflammatory conditions (Lin et al., 2019b; Song and Long, 2020). In our research, significantly elevated ROS content was detected in diabetic conditions, in which the expression levels of NRF2 and GPX4 were both diminished, suggesting reduced antioxidative capacity and an activated ferroptosis process. However, these issues were rescued by treatment with either P2Y_14_ shRNA or P2Y_14_-targeted naringin, demonstrating that ferroptosis may become involved in DCAN progression via the P2Y_14_ receptor and that P2Y_14_-targeted naringin can mitigate DCAN by boosting the NRF2/GPX4 pathway, eliminating ROS and thus inhibiting the ferroptosis process.

In our results, there are several issues that require more attention. First, double bands were shown in the Western blotting image of NRF2, which was also observed by other scientists and published in the research paper entitled “Prevention of Carcinogen-Induced Oral Cancer by Sulforaphane” (Bauman et al., 2016). We think NRF2 is involved in abundant interactions with various molecules, such as KEAP1 molecules and NRF2 inducers, by which it can sensitively regulate the expression of cytoprotective proteins, such as GPX4 (Tonelli et al., 2018; Baird and Yamamoto, 2020). Therefore, the band of NRF2 will be slightly affected and will vary in different cases of oxidative stress, thus several bands may be shown. It was also a surprising phenomenon that the level of ROS (a general indicator of ferroptosis) in the DM + P2Y_14_ shRNA group and DM + naringin group was even lower than that in the control group, which was also similarly demonstrated in another paper entitled “Inhibition of ferroptosis by upregulating NRF2 delayed the progression of diabetic nephropathy” (Li et al., 2021). In this paper, it was suggested that compared with those in the control group, the levels of MDA and iron (the other indicators of ferroptosis) were extremely diminished after the rescue of ferroptosis by fenofibrate. There are many possibilities and hypotheses based on these results, and we suppose that naringin can affect various pathways of ferroptosis and interact with several receptors in SCG to achieve a powerful anti-inflammatory effect; thus, the ROS level becomes even lower than that in the control group. Additional research is required to test the hypotheses mentioned above.

The paper has some limitations. One limitation in our research is the lack of real-time experiments to accurately assess the interaction between naringin and the P2Y_14_ receptor, although molecular docking provided some evidence. One paper entitled “Fragment screening by SPR and advanced application to GPCRs” gave us some insight (Shepherd et al., 2014). The interaction can be measured by detecting the change in refractive index in real time at the surface interface where a binding event occurs between the receptors and drugs, by which detailed information of the binding, including drug affinity, kinetics and thermodynamics can be deeply understood (Shepherd et al., 2014). Moreover, we primarily explored the ferroptosis involved in DCAN by several key and preliminary experiments, but more scientific research is required to help us better understand it.

In conclusion, diabetes can trigger SGC activation mediated by the P2Y_14_ receptor under oxidative stress conditions, followed by autonomic neuropathy and cardiac damage. Significantly, P2Y_14_ is prominently overexpressed, inflammatory factors (such as IL-1β) are massively released and gap junctions between nerve cells are formed in diabetic cases, all of which facilitate DCAN, consequently causing cardiac damage. Our research also demonstrated that the GPX4/NRF2 pathway was suppressed, and thus, ROS accumulated in the case of DCAN. However, naringin, a traditional Chinese medicine, that targets P2Y_14_ receptors, can not only diminish the expression of the P2Y_14_ receptor and inflammatory factors but also restore the function of the antioxidant GPX4/NRF2 pathway, thus effectively relieving DCAN.

Our research preliminarily demonstrated the potent alleviative effect of naringin on DCAN as well as one possible mechanism, which may provide a promising perspective for DCAN treatment in the future.

## Data Availability

The original contributions presented in the study are included in the article/supplementary material, further inquiries can be directed to the corresponding author.
