# Supplementary material for: Naringin Relieves Diabetic Cardiac Autonomic Neuropathy Mediated by P2Y14 Receptor in Superior Cervical Ganglion
Source: Front Pharmacol. 2022 Apr 21;13:873090. doi: 10.3389/fphar.2022.873090 (PMC9068893; doi:10.3389/fphar.2022.873090)
Supplement: Supplementary file 1 [file Presentation1.pptx]

## Slide 1
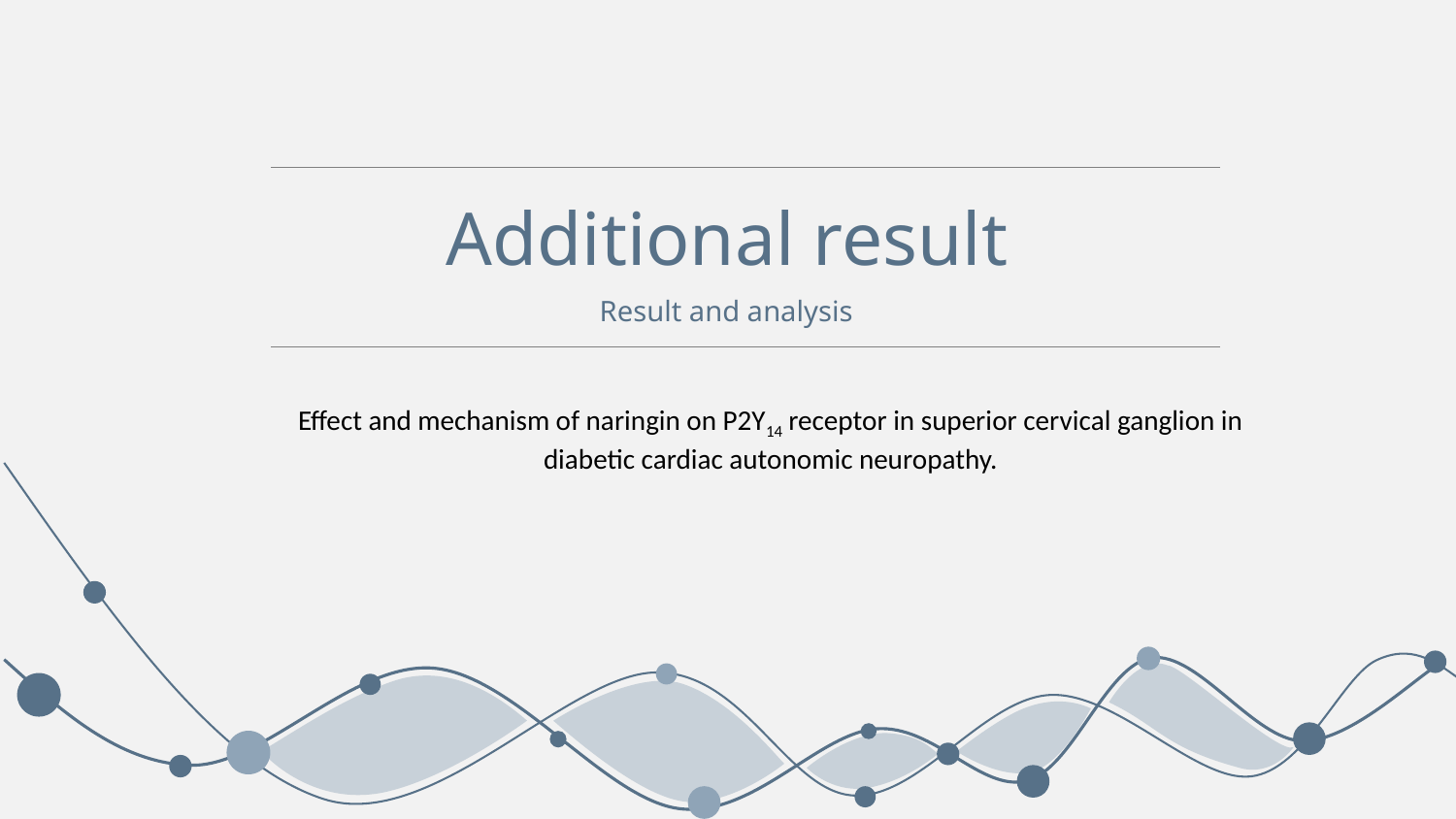

Additional result
Result and analysis
Effect and mechanism of naringin on P2Y14 receptor in superior cervical ganglion in diabetic cardiac autonomic neuropathy.

## Slide 2
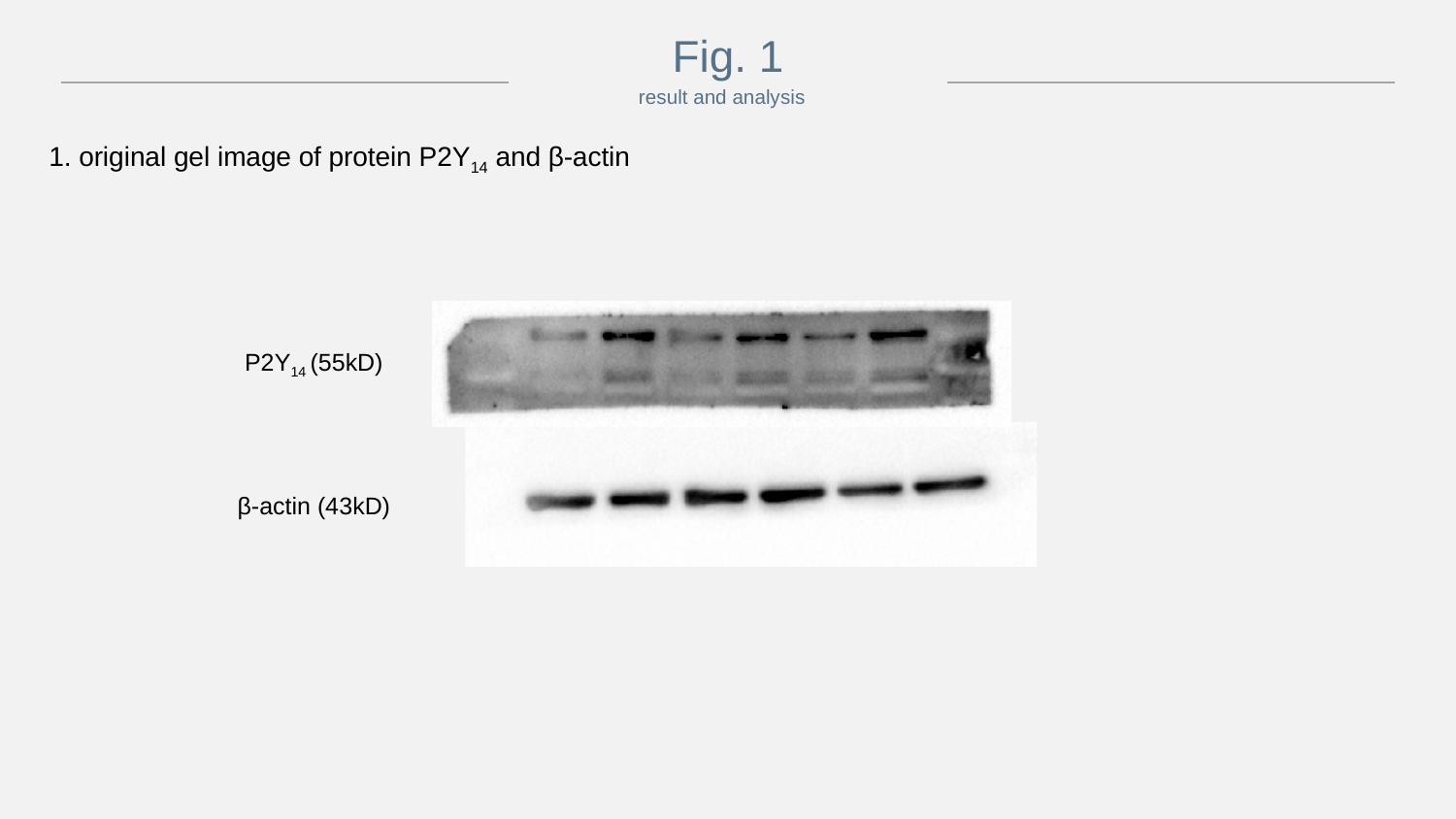

Fig. 1
result and analysis
1. original gel image of protein P2Y14 and β-actin
P2Y14 (55kD)
β-actin (43kD)

## Slide 3
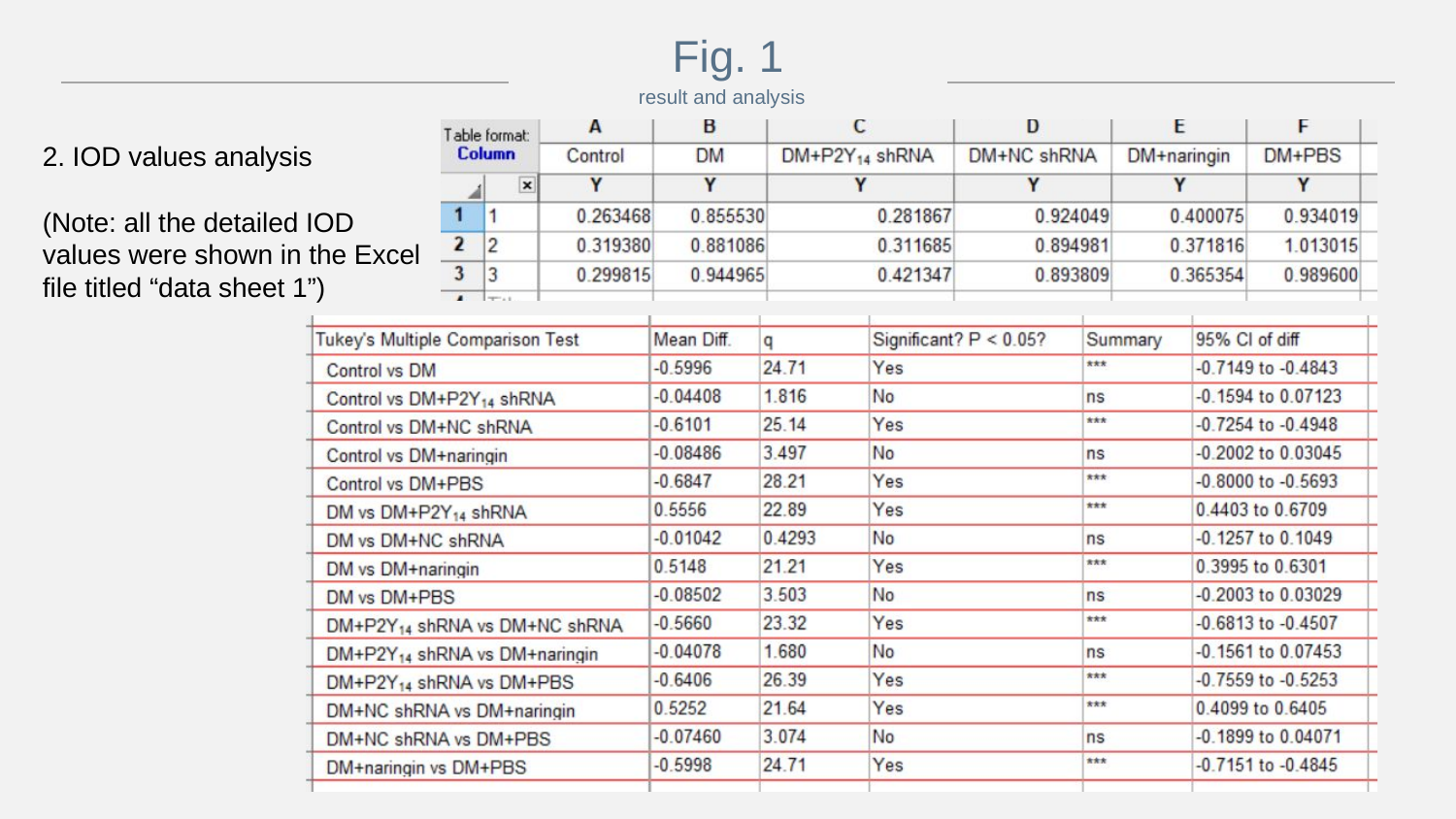

Fig. 1
result and analysis
2. IOD values analysis
(Note: all the detailed IOD values were shown in the Excel file titled “data sheet 1”)

## Slide 4
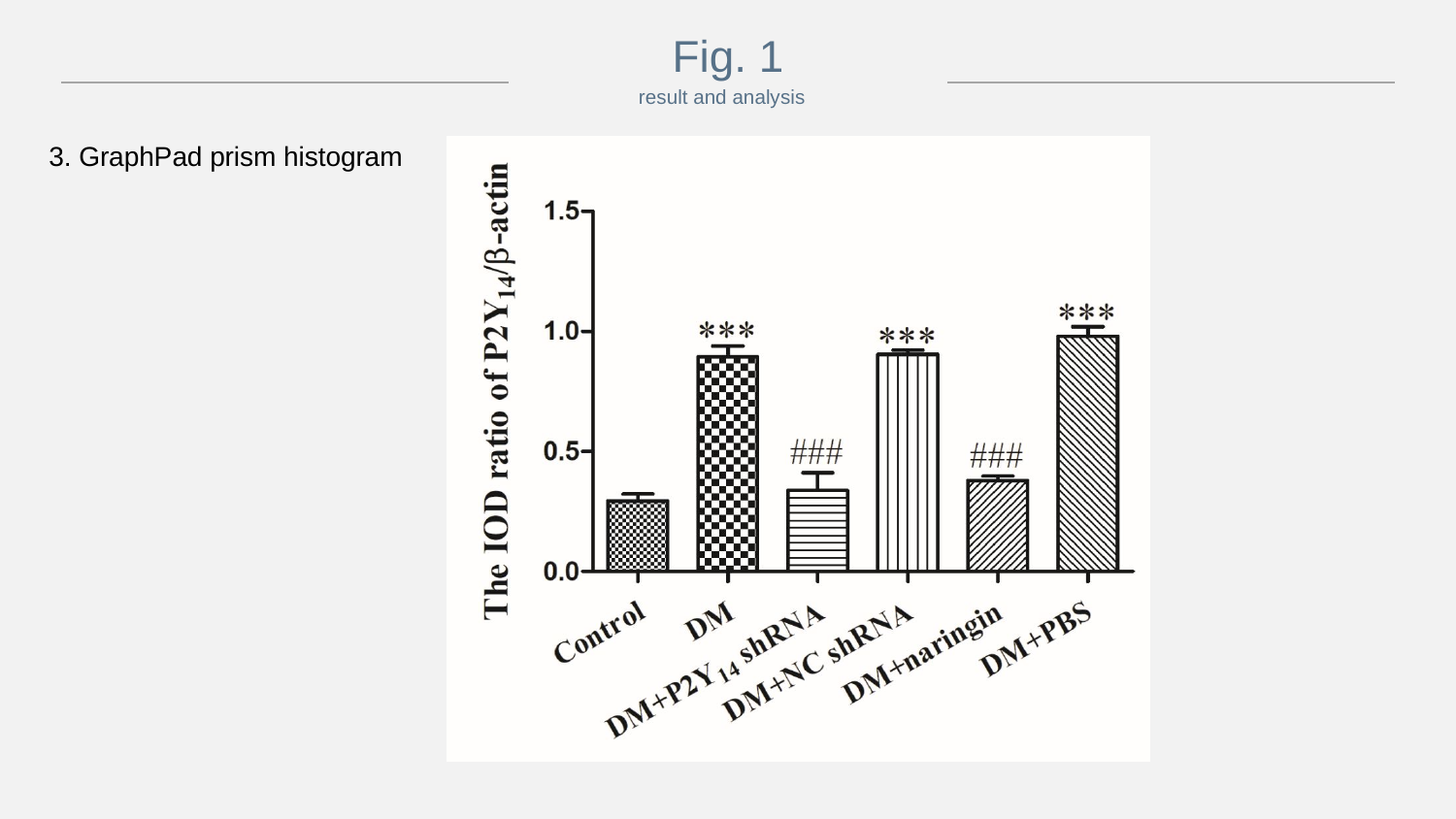

Fig. 1
result and analysis
3. GraphPad prism histogram

## Slide 5
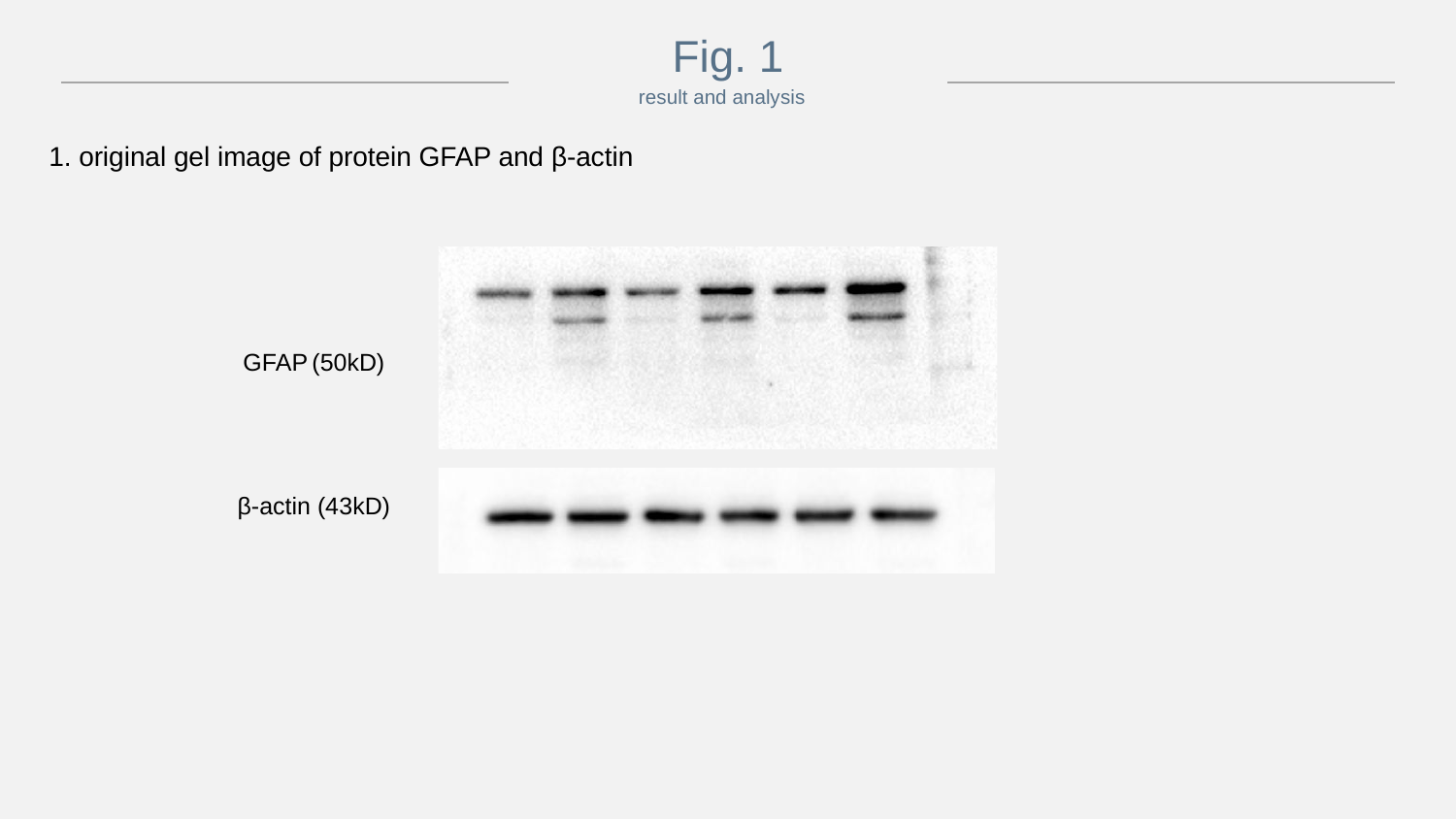

Fig. 1
result and analysis
1. original gel image of protein GFAP and β-actin
GFAP (50kD)
β-actin (43kD)

## Slide 6
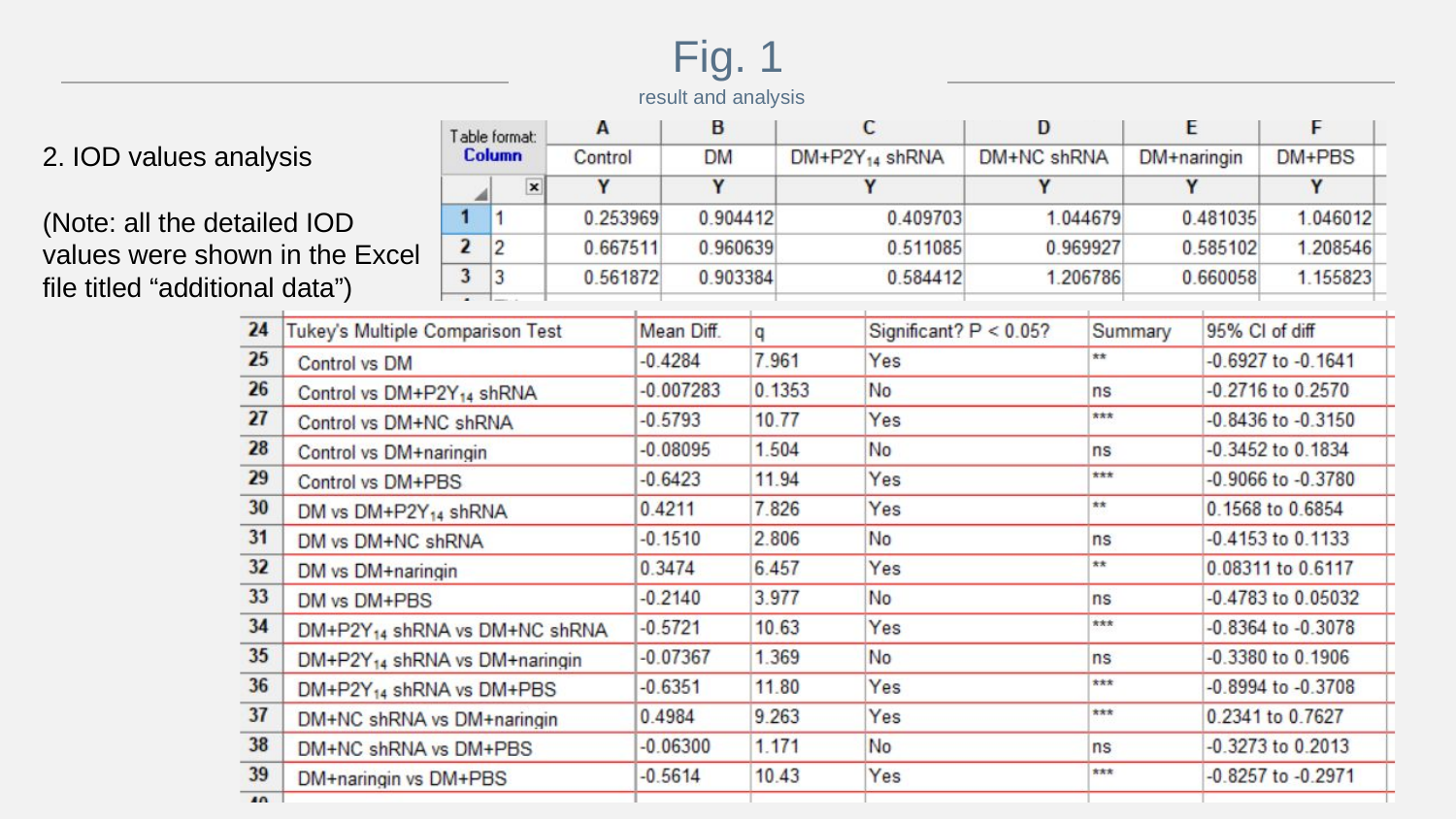

Fig. 1
result and analysis
2. IOD values analysis
(Note: all the detailed IOD values were shown in the Excel file titled “additional data”)

## Slide 7
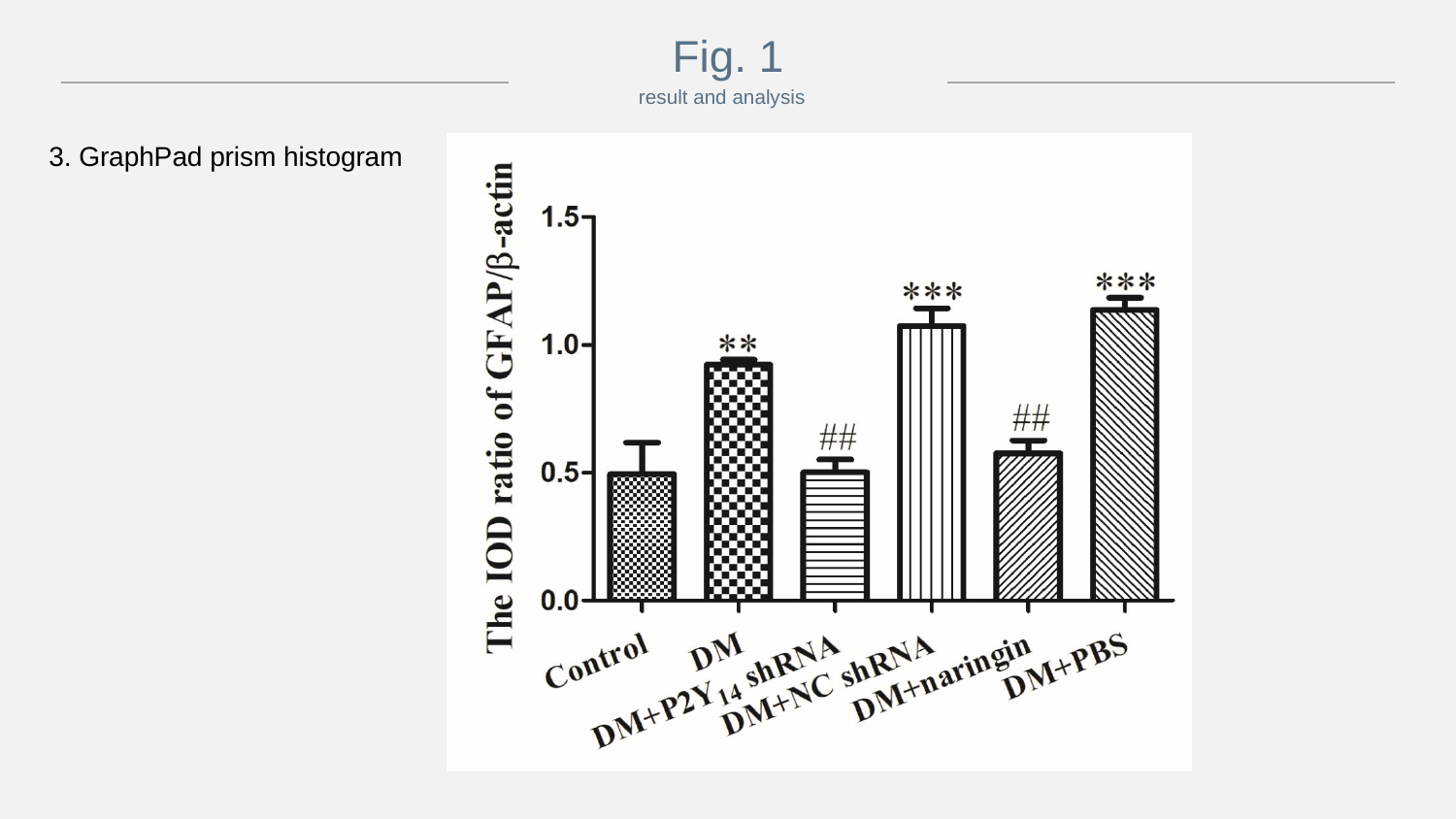

Fig. 1
result and analysis
3. GraphPad prism histogram

## Slide 8
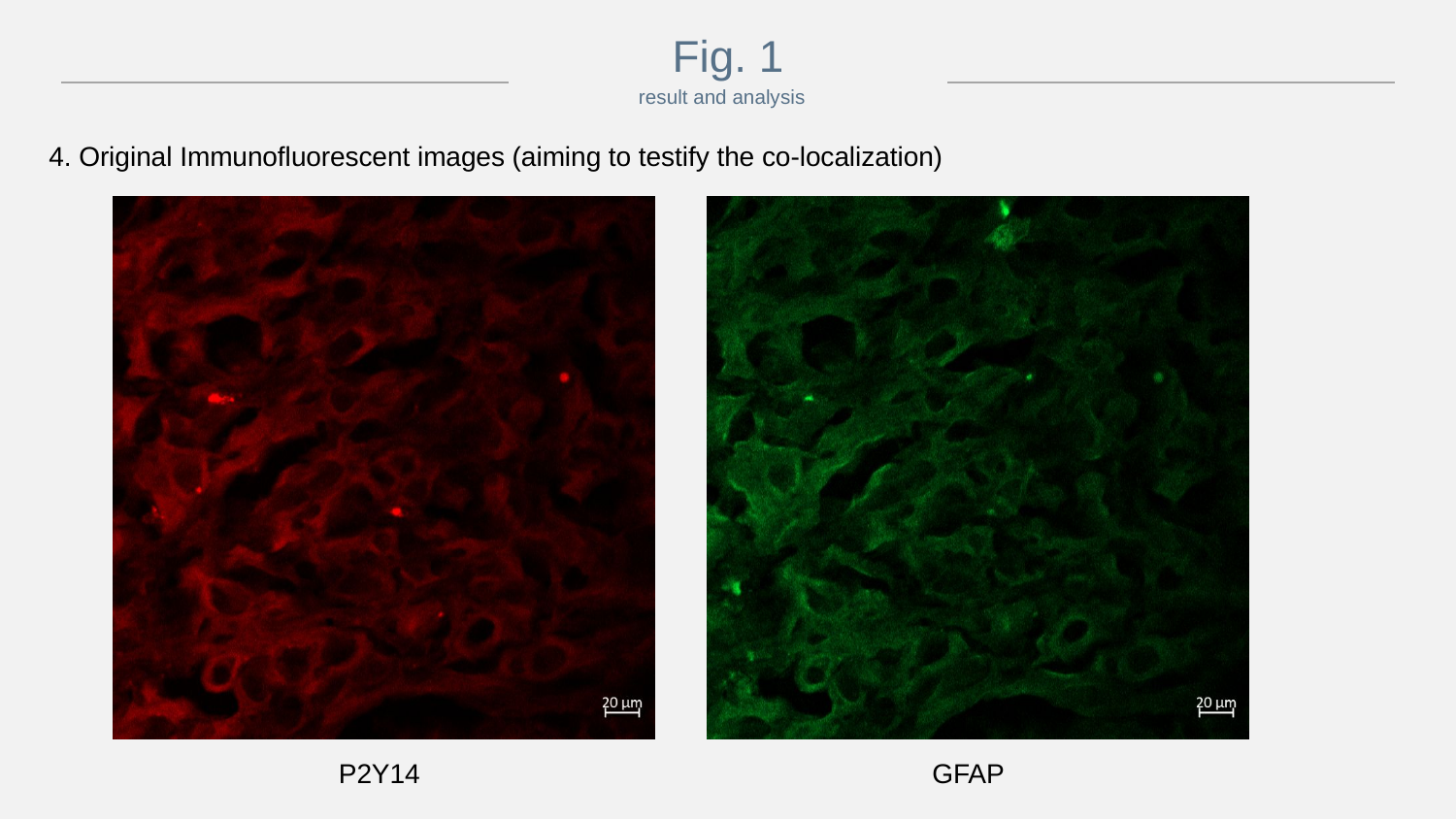

Fig. 1
result and analysis
4. Original Immunofluorescent images (aiming to testify the co-localization)
P2Y14
GFAP

## Slide 9
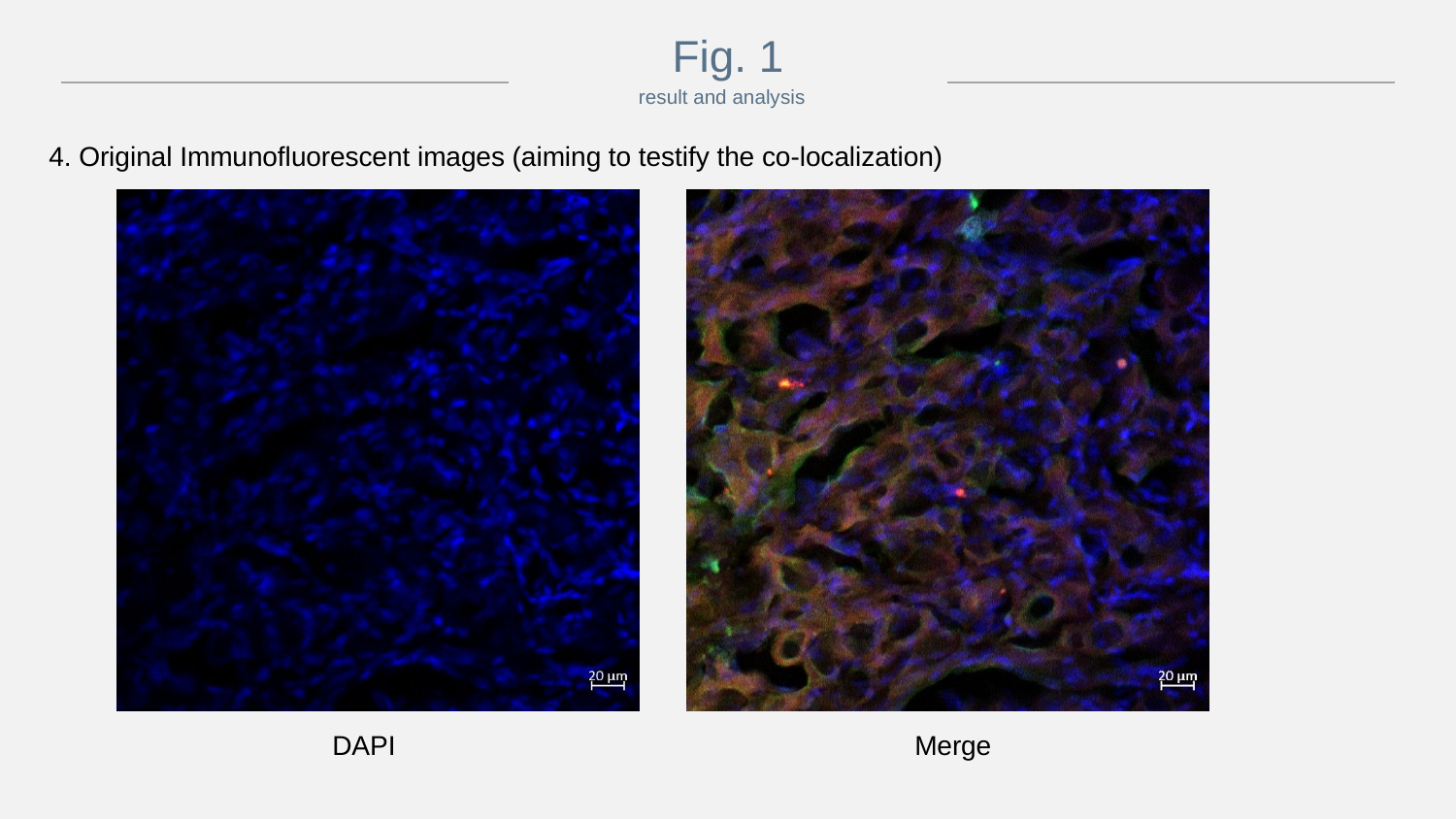

Fig. 1
result and analysis
4. Original Immunofluorescent images (aiming to testify the co-localization)
Merge
DAPI

## Slide 10
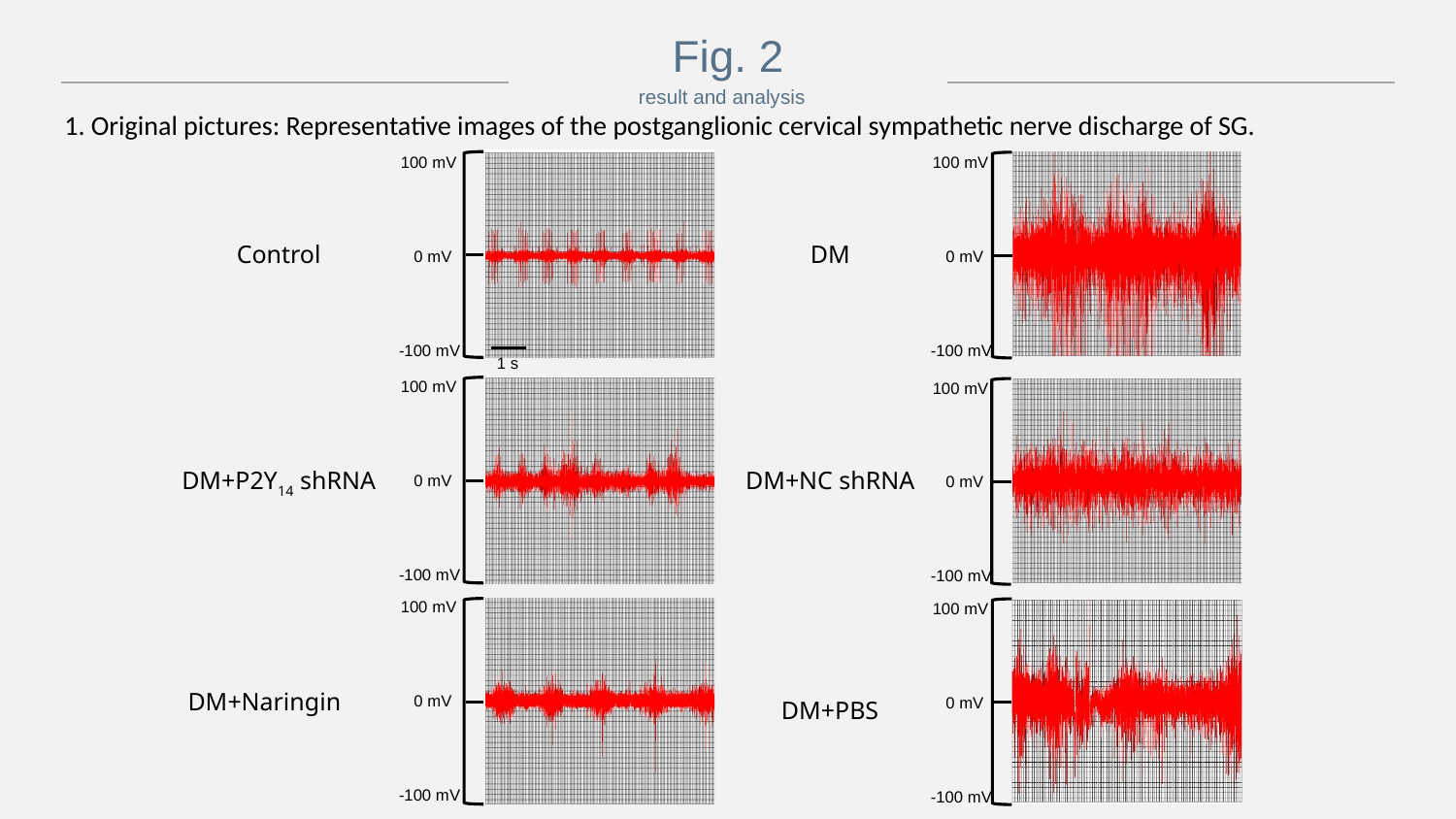

Fig. 2
result and analysis
1. Original pictures: Representative images of the postganglionic cervical sympathetic nerve discharge of SG.
100 mV
0 mV
-100 mV
100 mV
0 mV
-100 mV
Control
DM
1 s
100 mV
0 mV
-100 mV
100 mV
0 mV
-100 mV
DM+P2Y14 shRNA
DM+NC shRNA
100 mV
0 mV
-100 mV
100 mV
0 mV
-100 mV
DM+Naringin
DM+PBS

## Slide 11
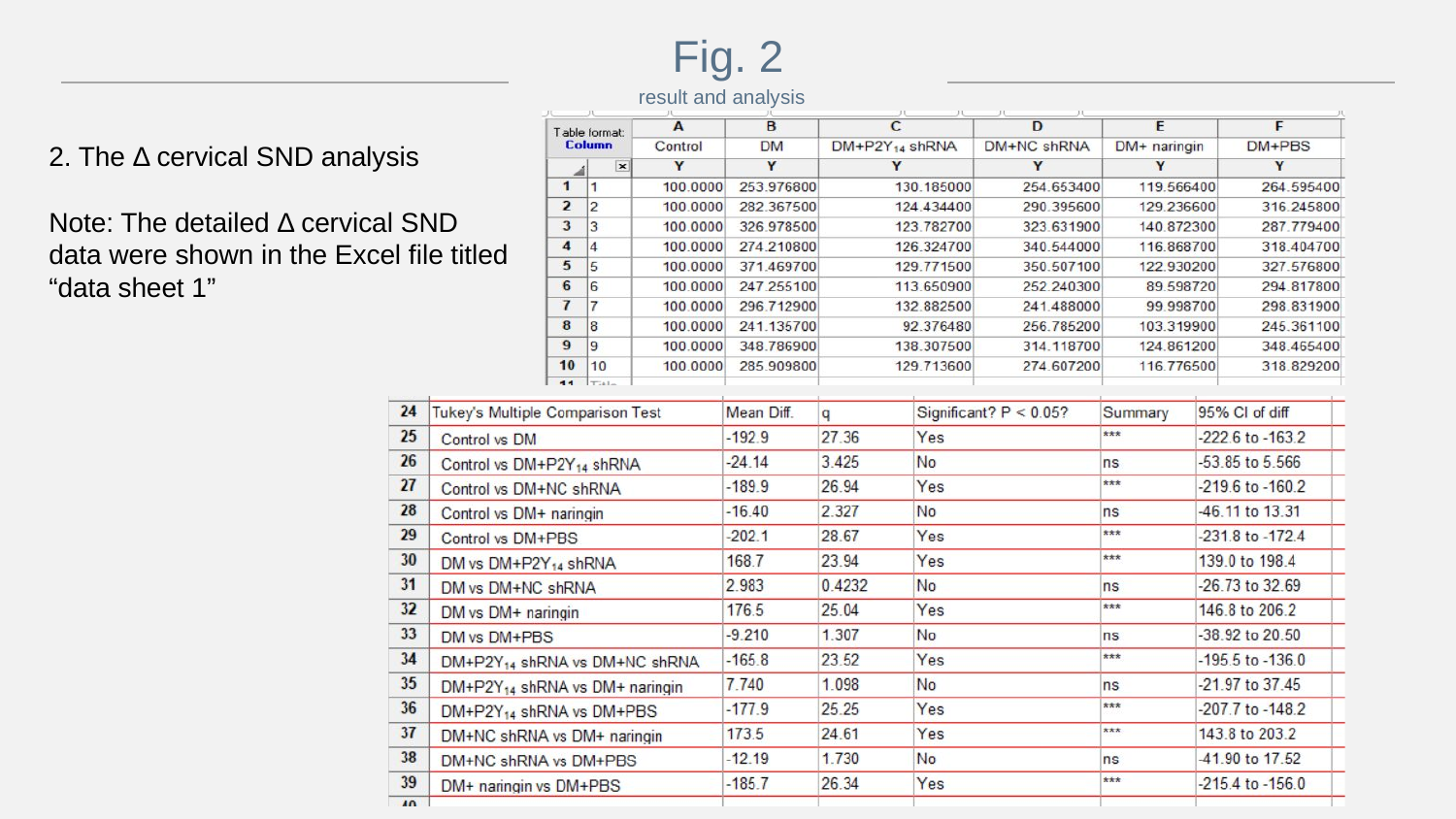

Fig. 2
result and analysis
2. The Δ cervical SND analysis
Note: The detailed Δ cervical SND data were shown in the Excel file titled “data sheet 1”

## Slide 12
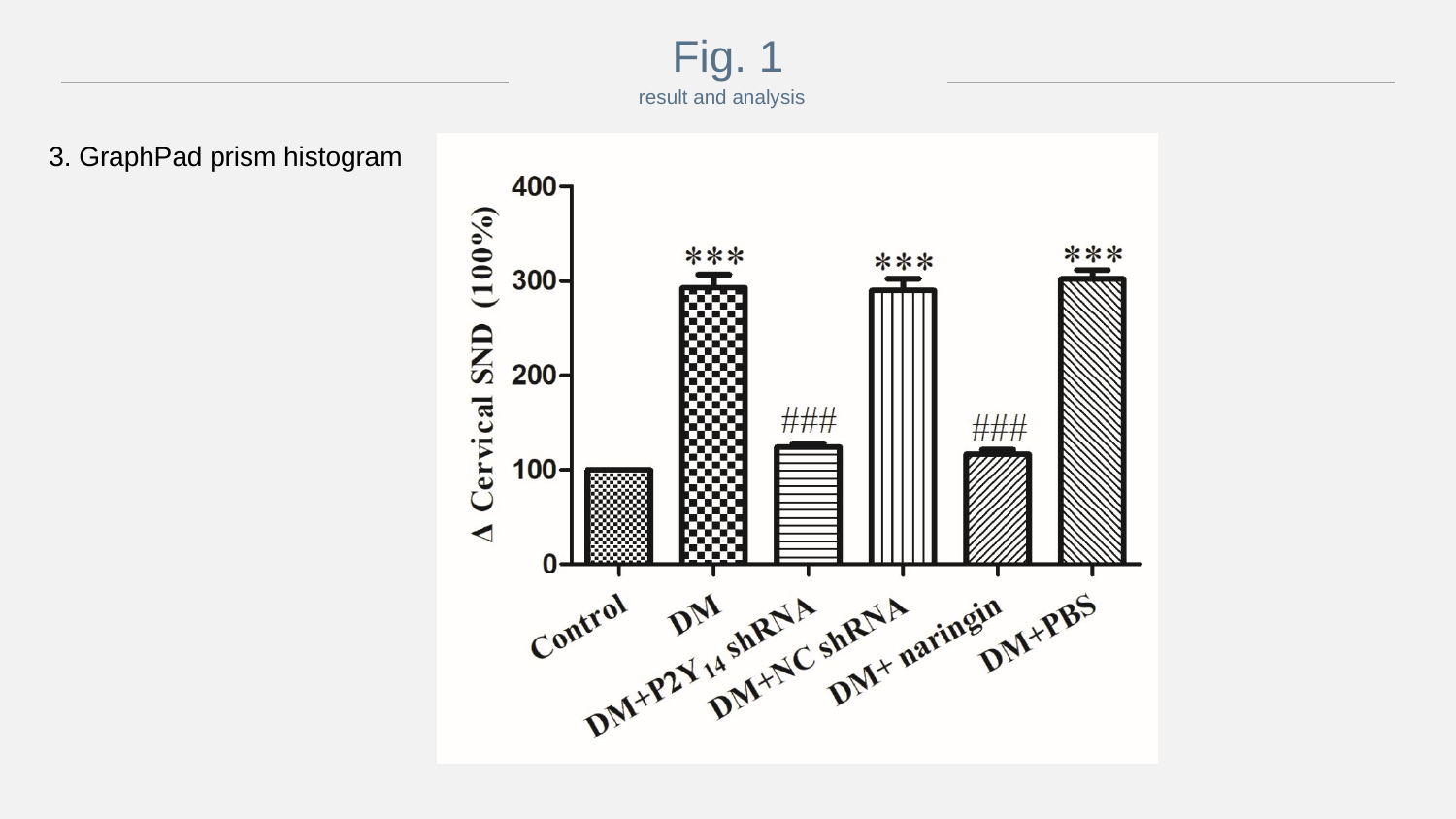

Fig. 1
result and analysis
3. GraphPad prism histogram

## Slide 13
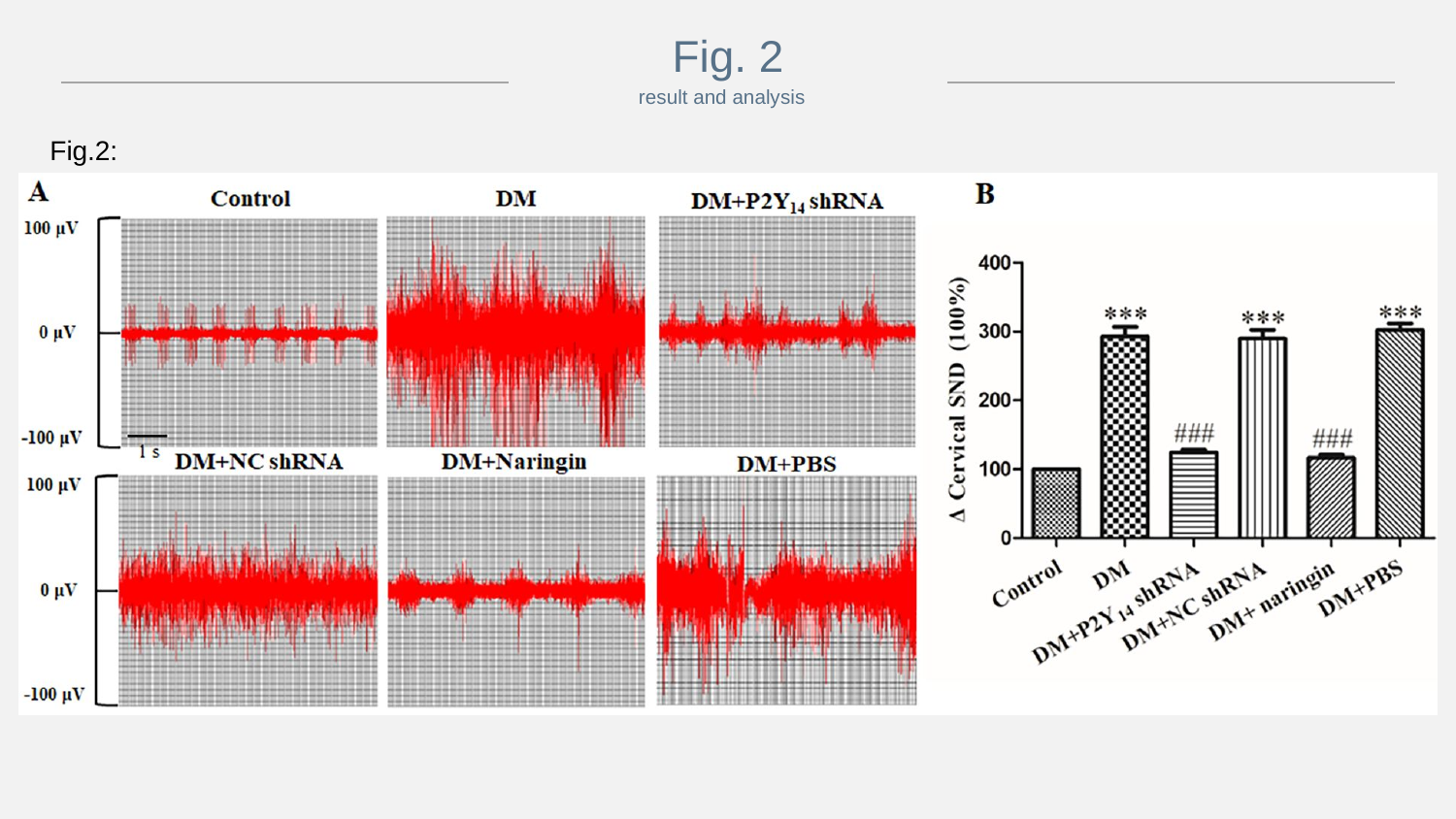

Fig. 2
result and analysis
Fig.2:

## Slide 14
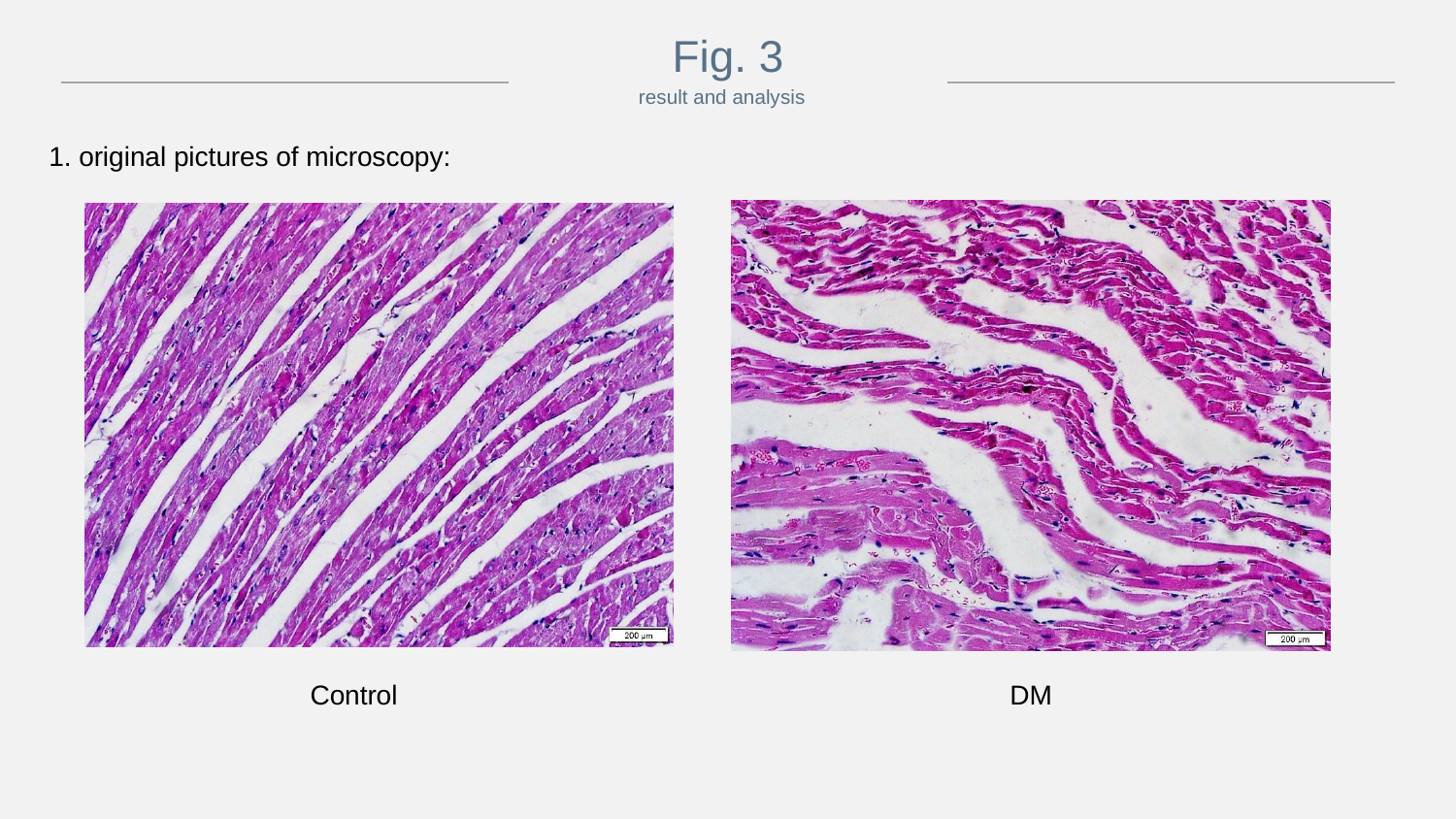

Fig. 3
result and analysis
1. original pictures of microscopy:
Control
DM

## Slide 15
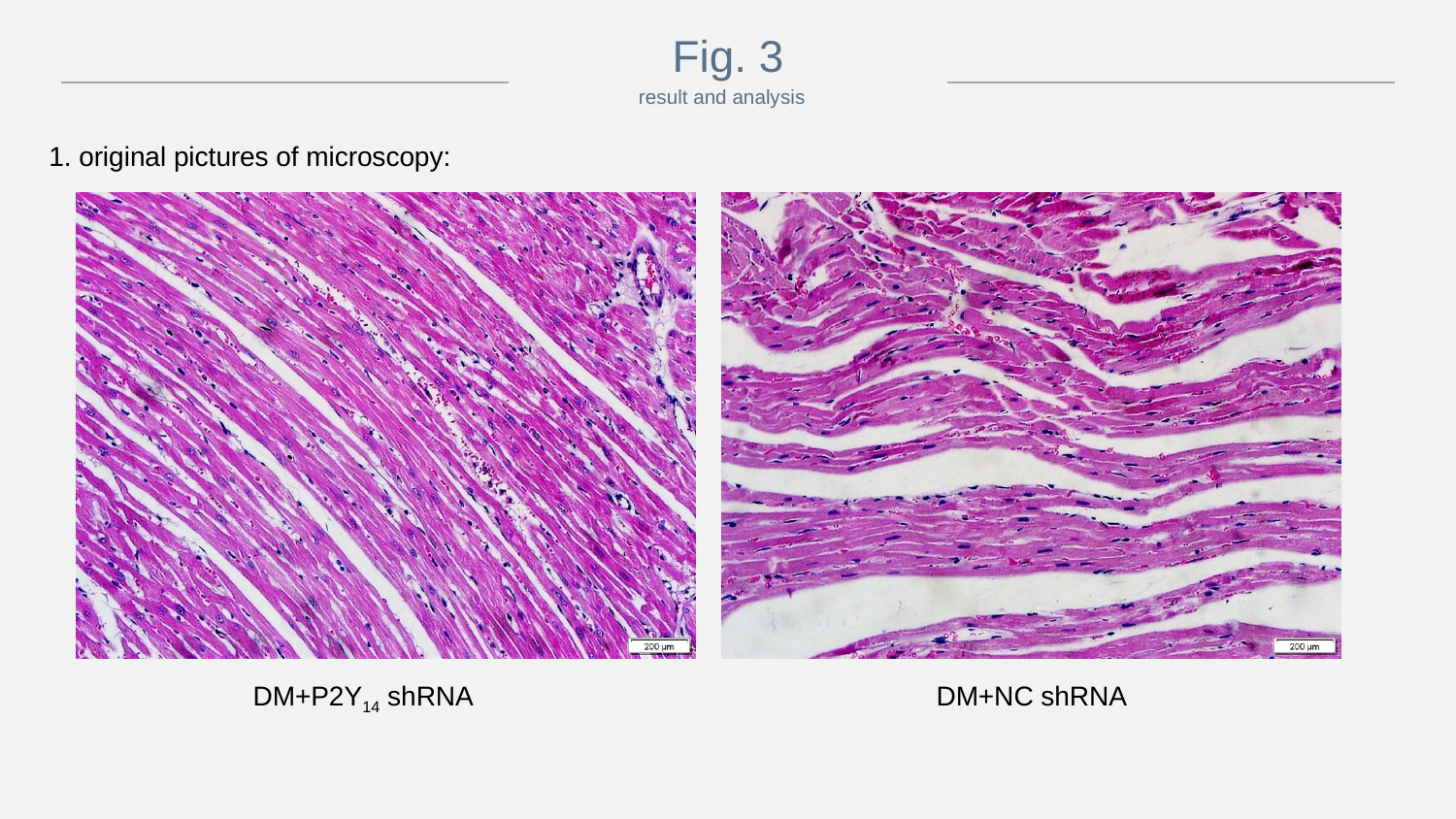

Fig. 3
result and analysis
1. original pictures of microscopy:
DM+P2Y14 shRNA
DM+NC shRNA

## Slide 16
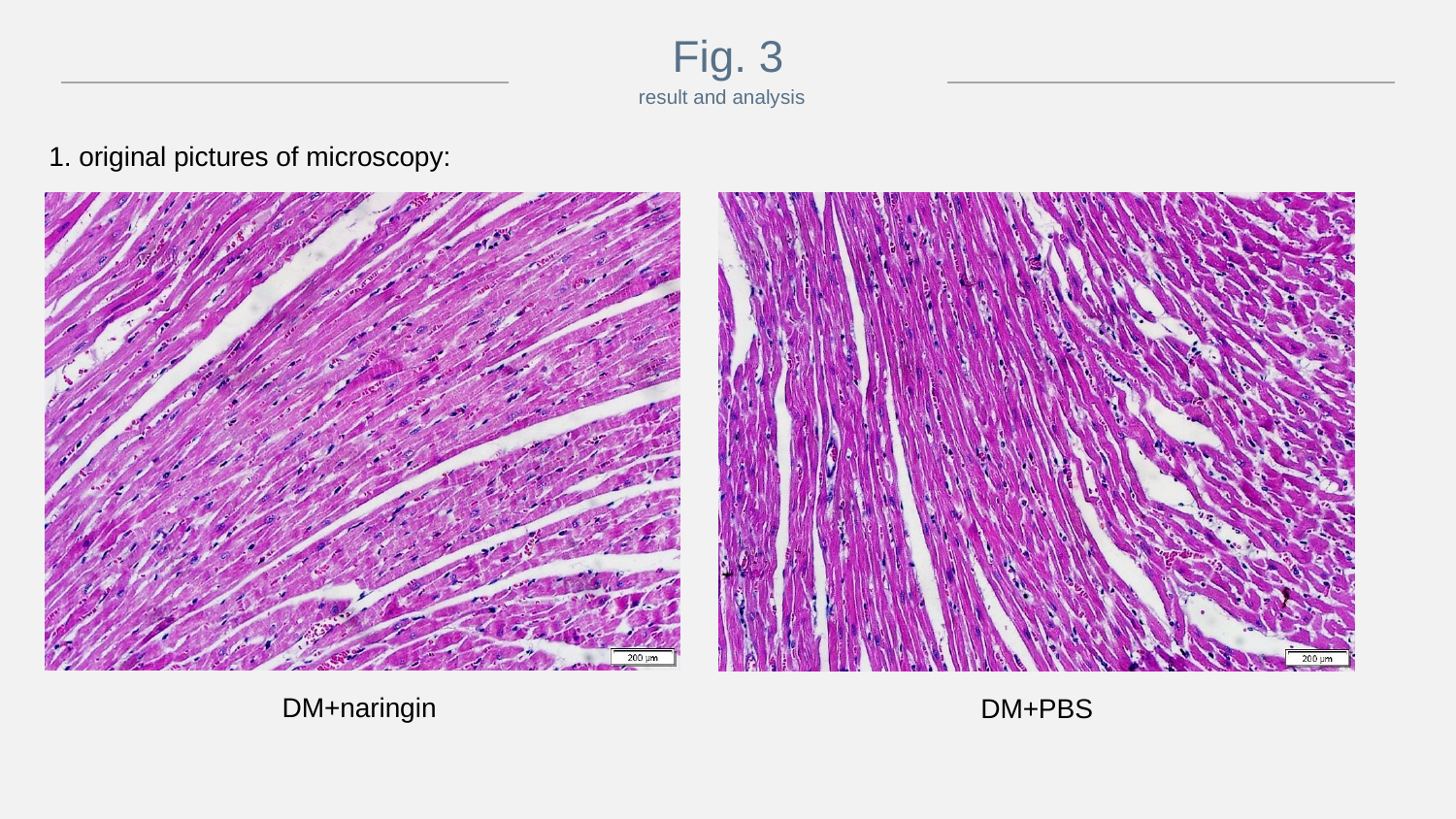

Fig. 3
result and analysis
1. original pictures of microscopy:
DM+naringin
DM+PBS

## Slide 17
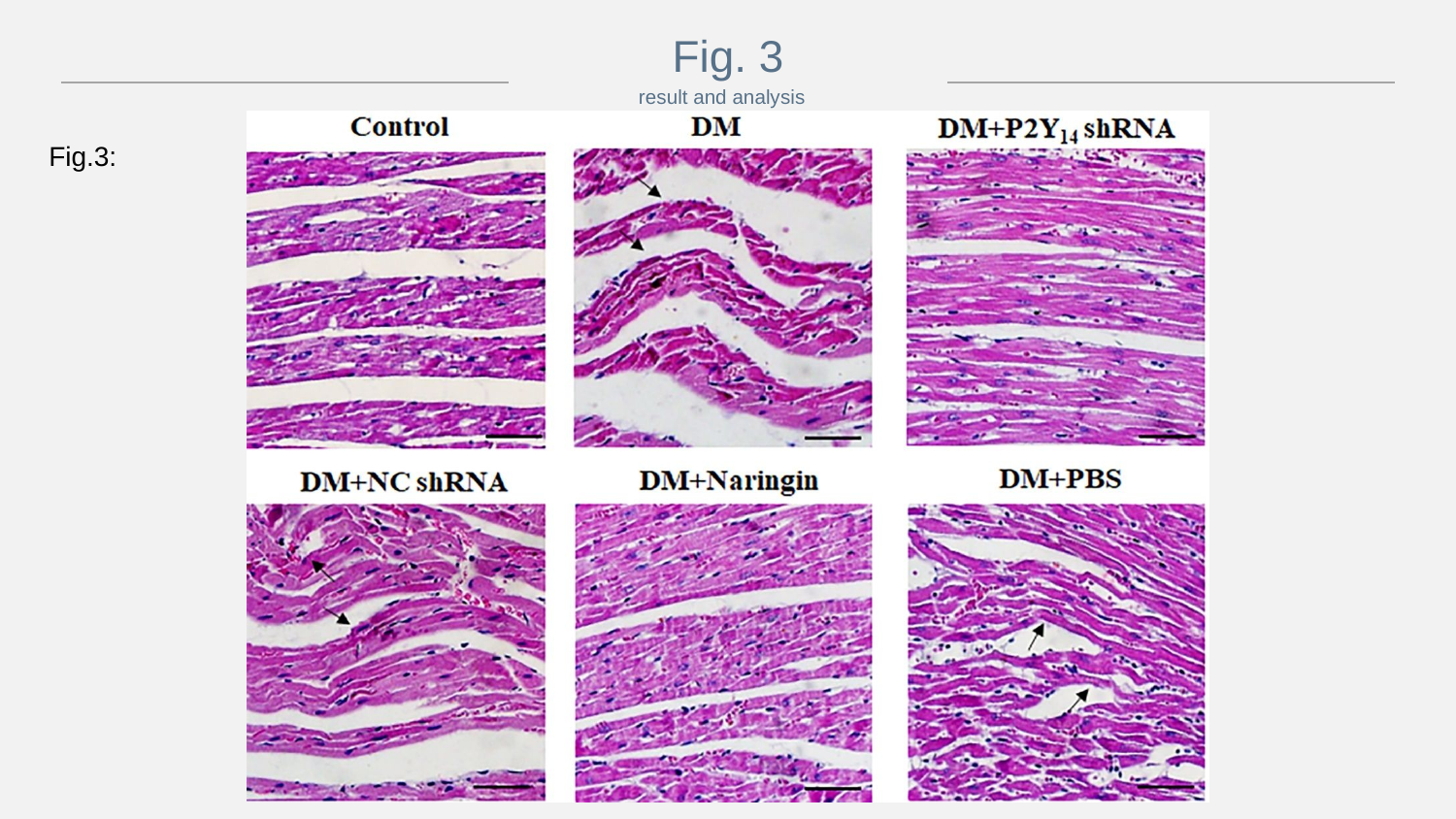

Fig. 3
result and analysis
Fig.3:

## Slide 18
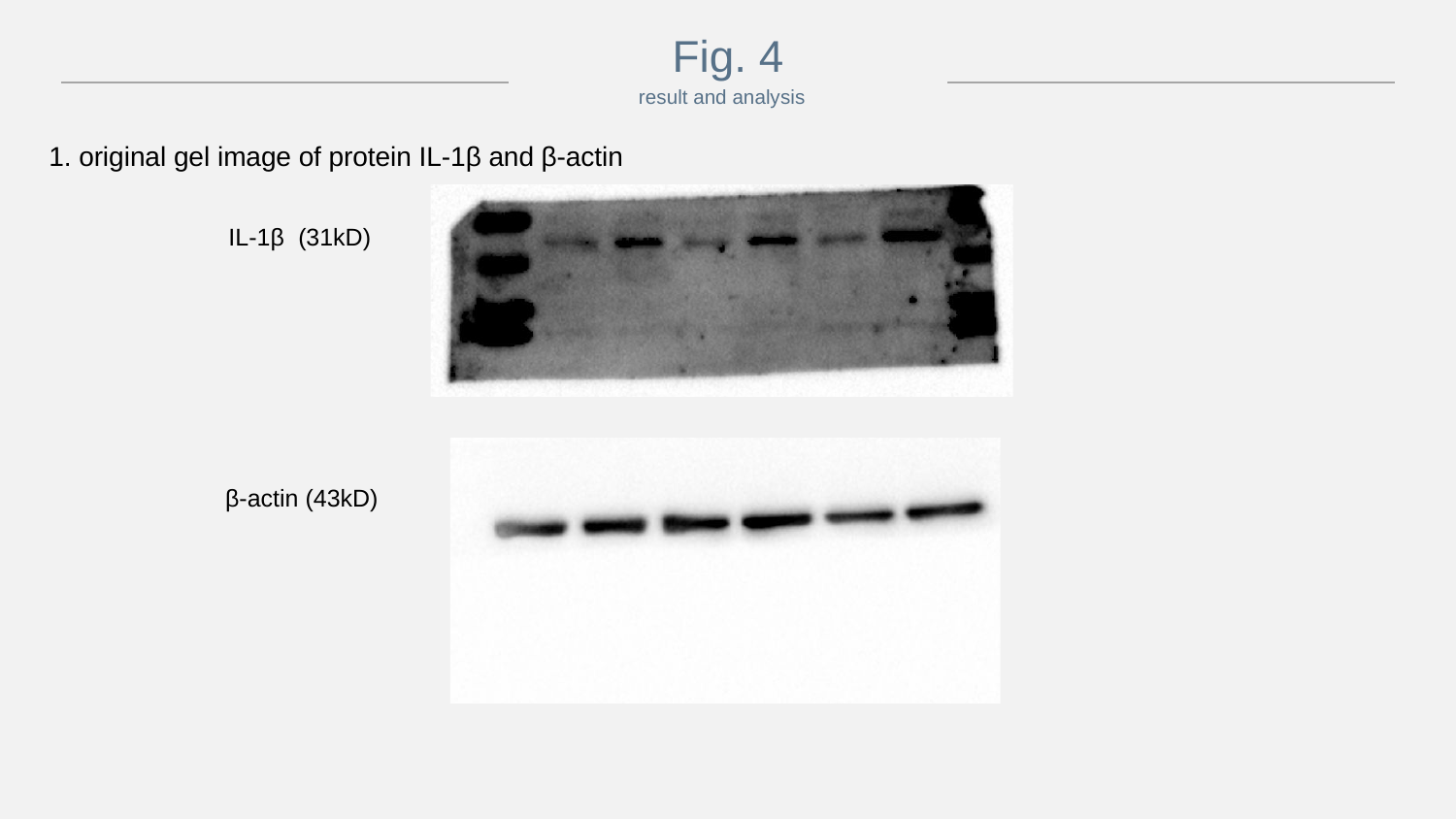

Fig. 4
result and analysis
1. original gel image of protein IL-1β and β-actin
IL-1β (31kD)
β-actin (43kD)

## Slide 19
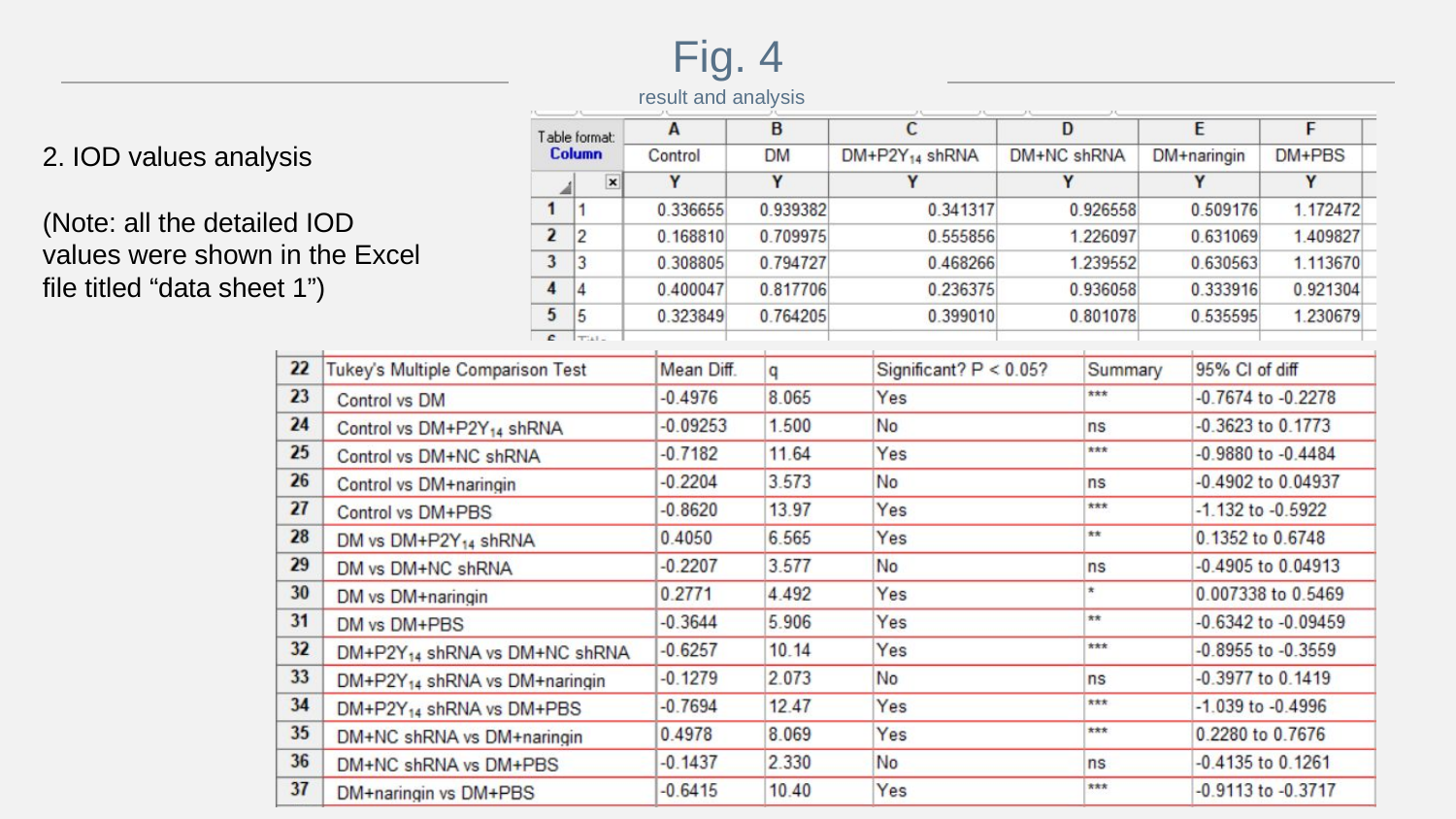

Fig. 4
result and analysis
2. IOD values analysis
(Note: all the detailed IOD values were shown in the Excel file titled “data sheet 1”)

## Slide 20
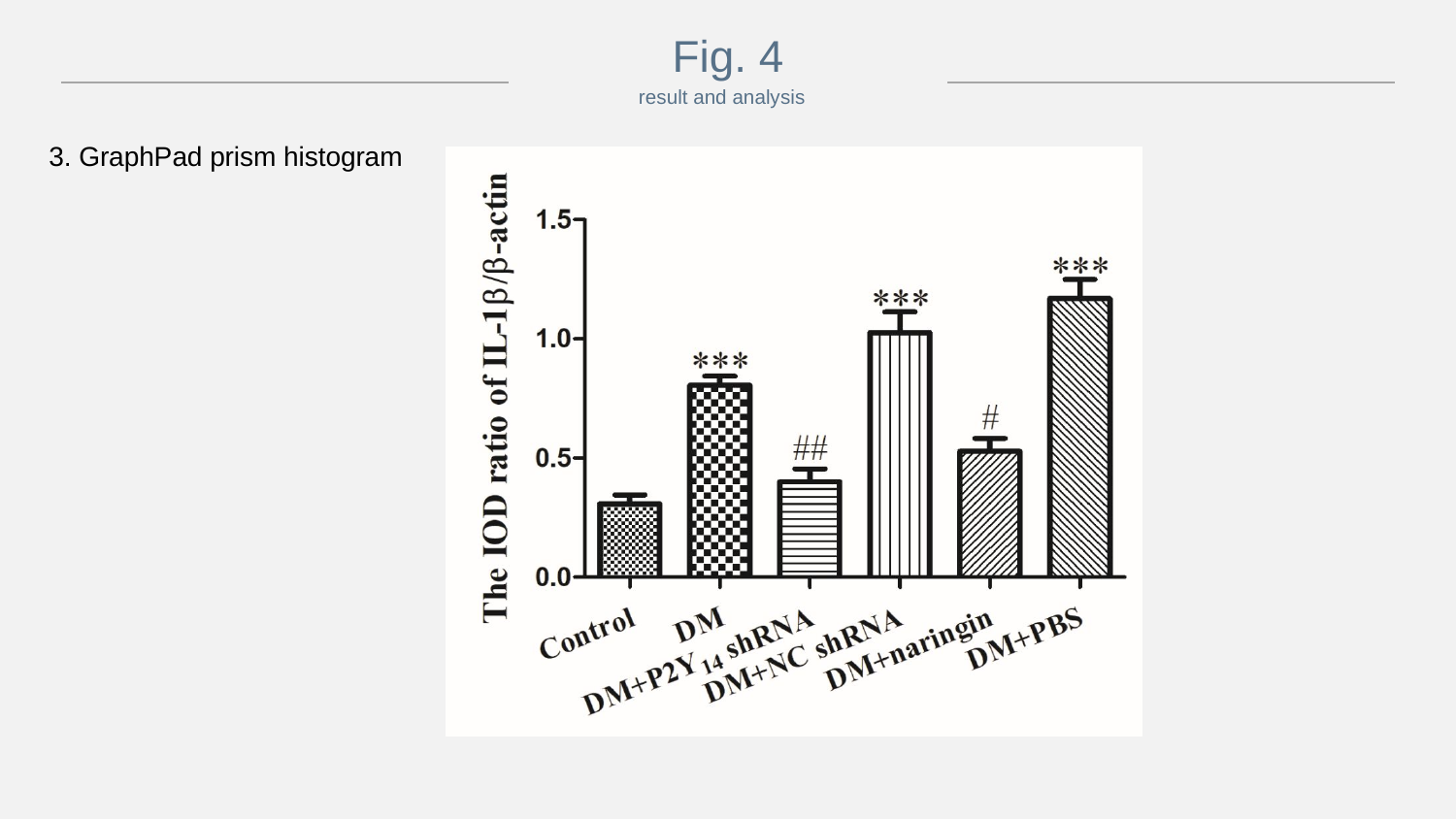

Fig. 4
result and analysis
3. GraphPad prism histogram

## Slide 21
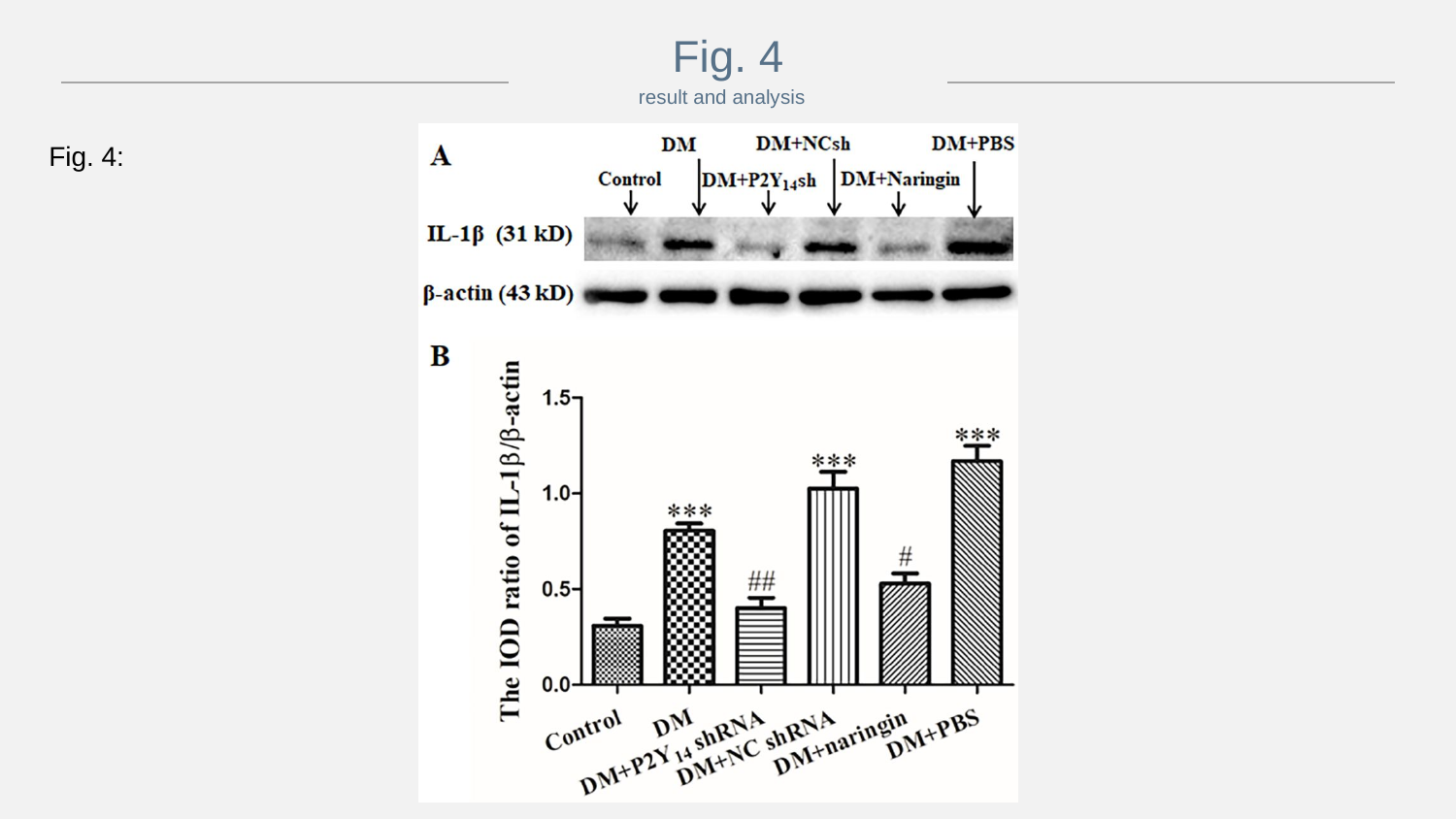

Fig. 4
result and analysis
Fig. 4:

## Slide 22
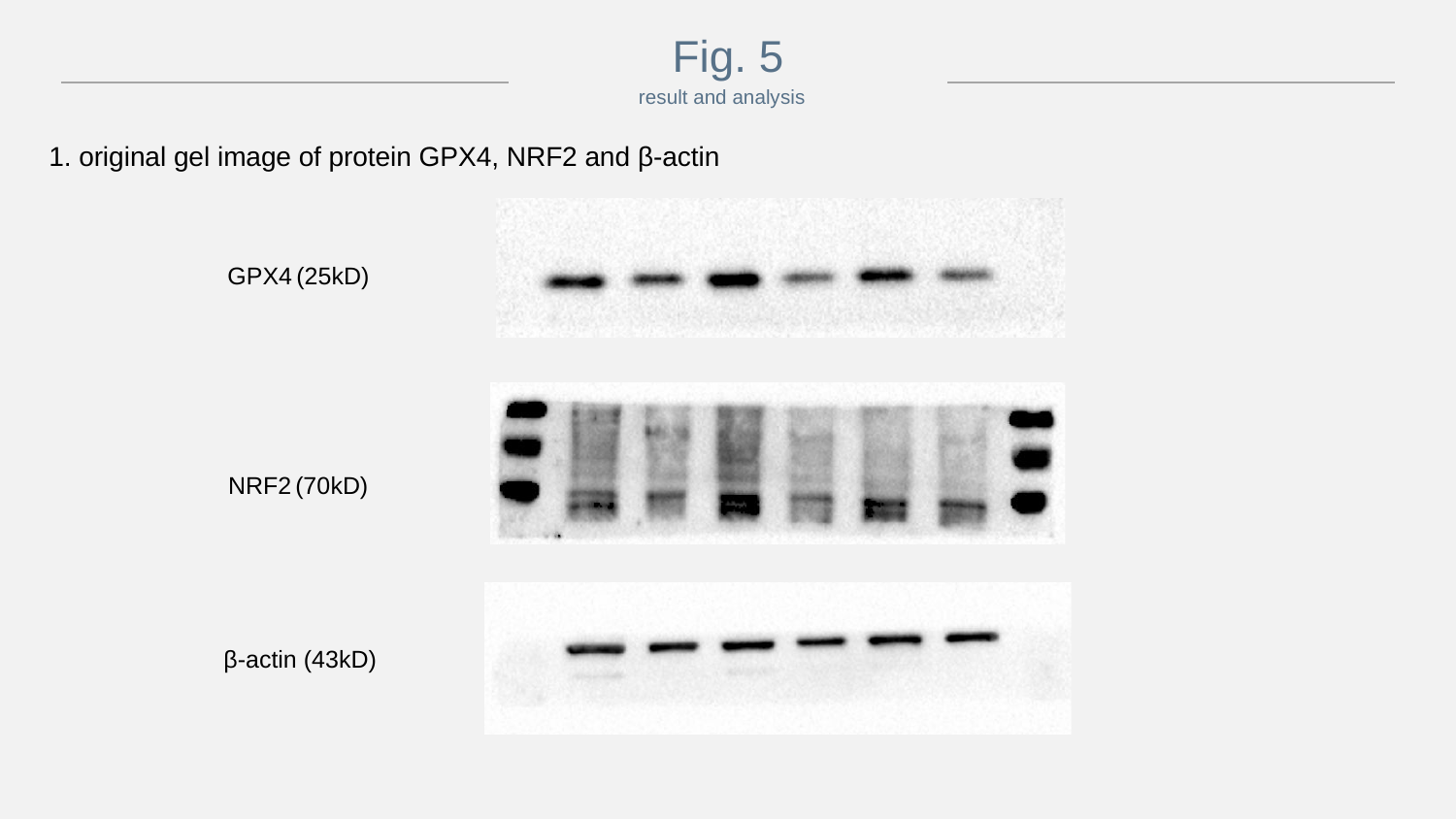

Fig. 5
result and analysis
1. original gel image of protein GPX4, NRF2 and β-actin
GPX4 (25kD)
NRF2 (70kD)
β-actin (43kD)

## Slide 23
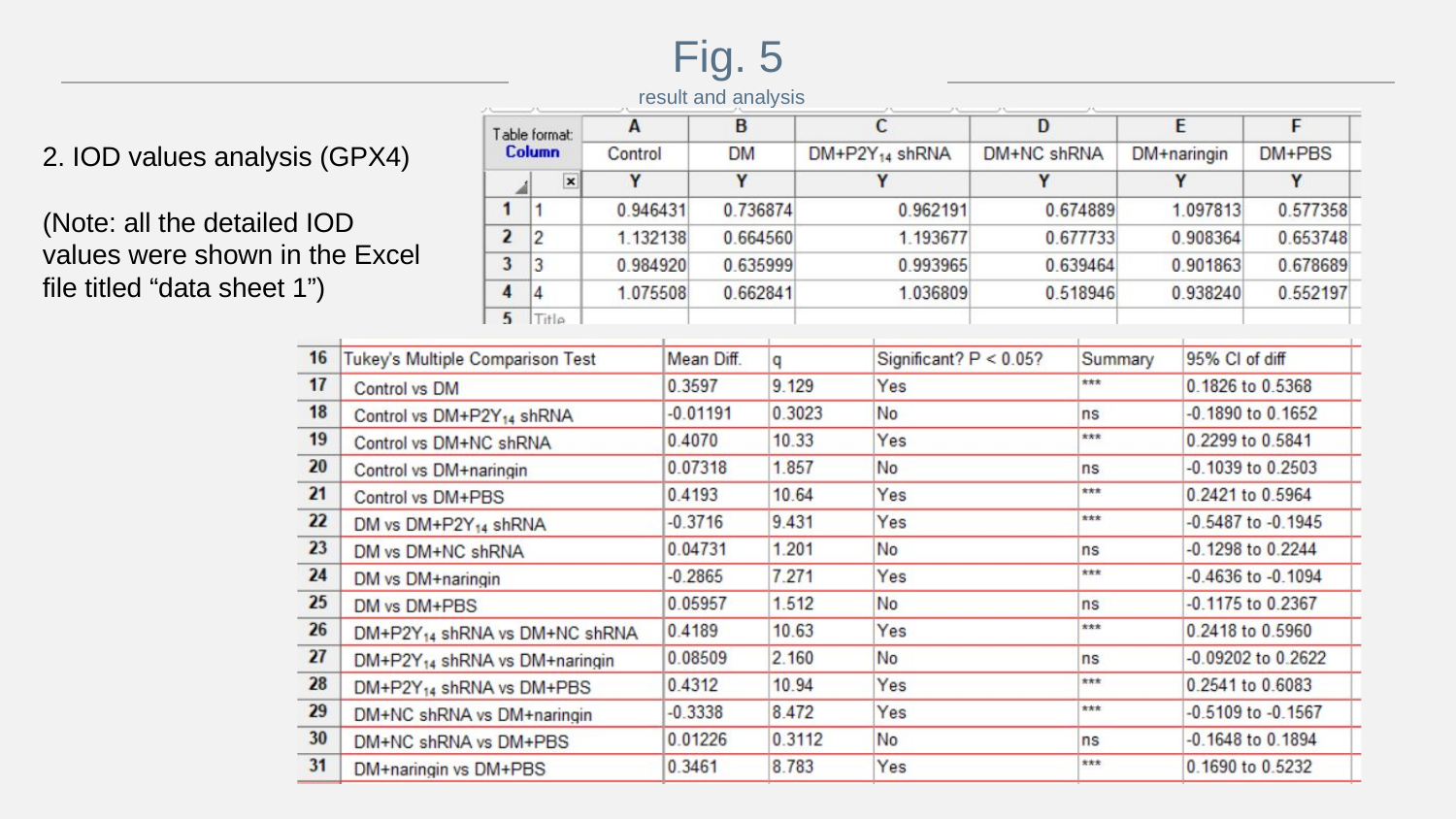

Fig. 5
result and analysis
2. IOD values analysis (GPX4)
(Note: all the detailed IOD values were shown in the Excel file titled “data sheet 1”)

## Slide 24
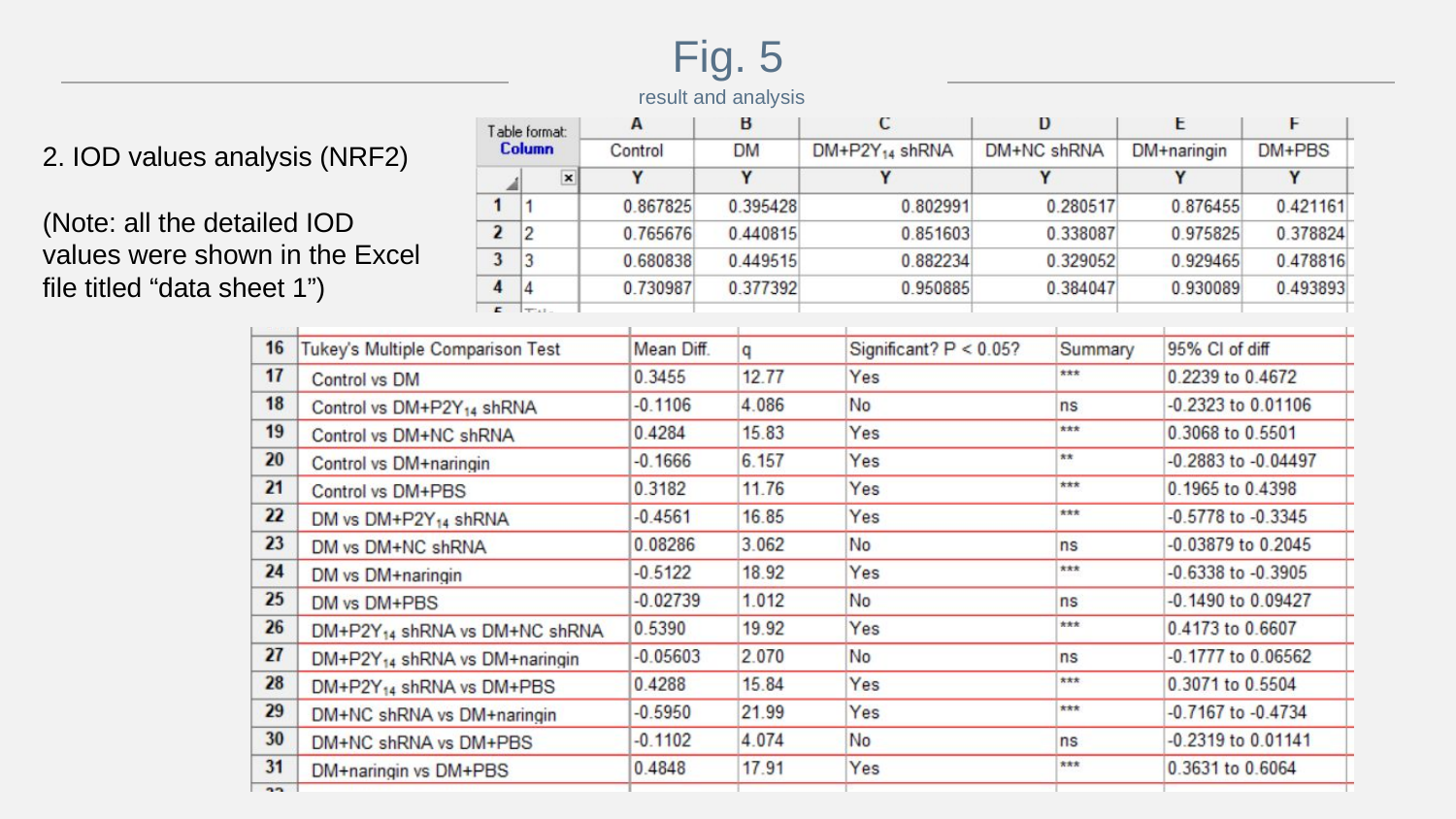

Fig. 5
result and analysis
2. IOD values analysis (NRF2)
(Note: all the detailed IOD values were shown in the Excel file titled “data sheet 1”)

## Slide 25
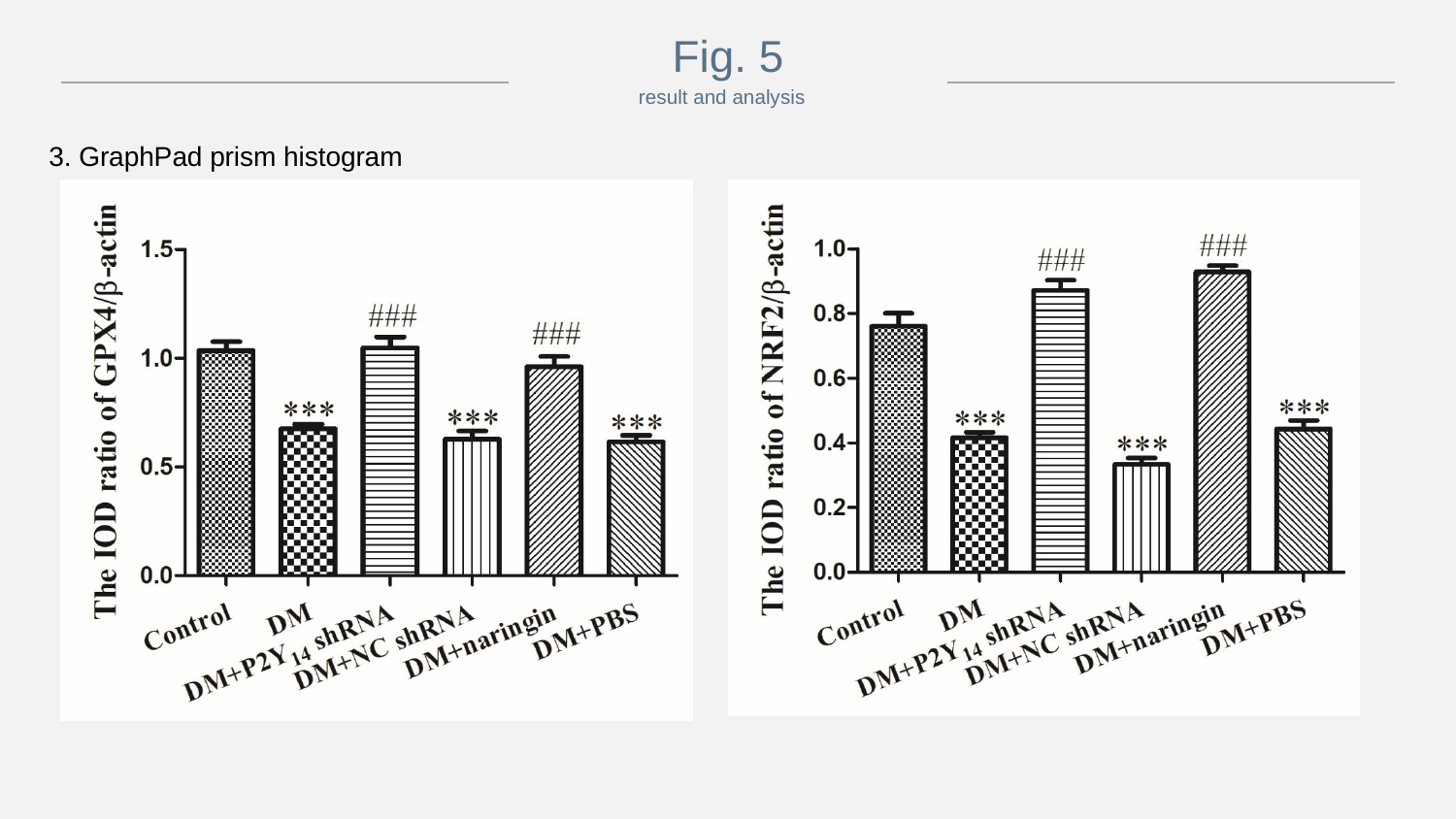

Fig. 5
result and analysis
3. GraphPad prism histogram

## Slide 26
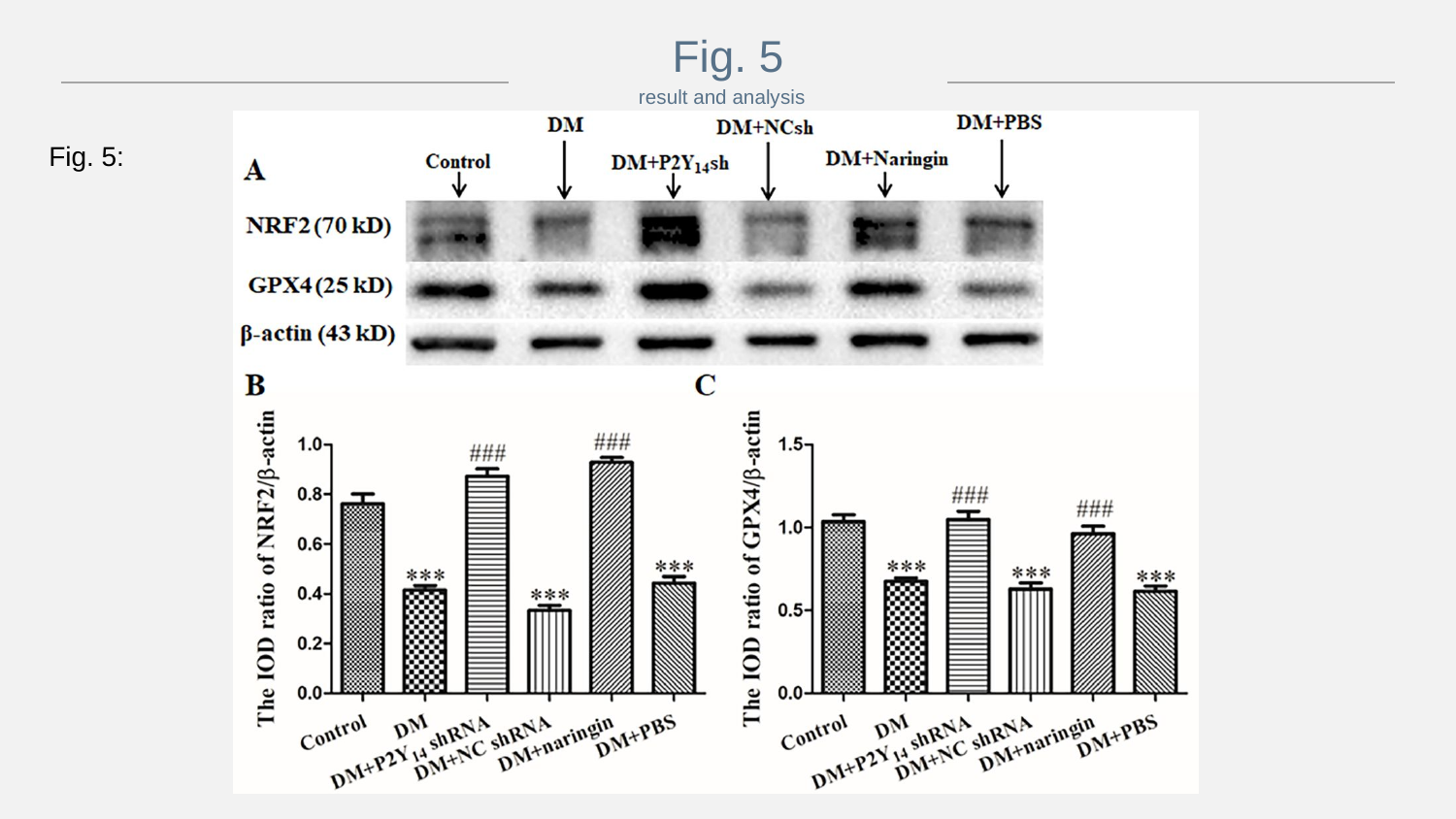

Fig. 5
result and analysis
Fig. 5:

## Slide 27
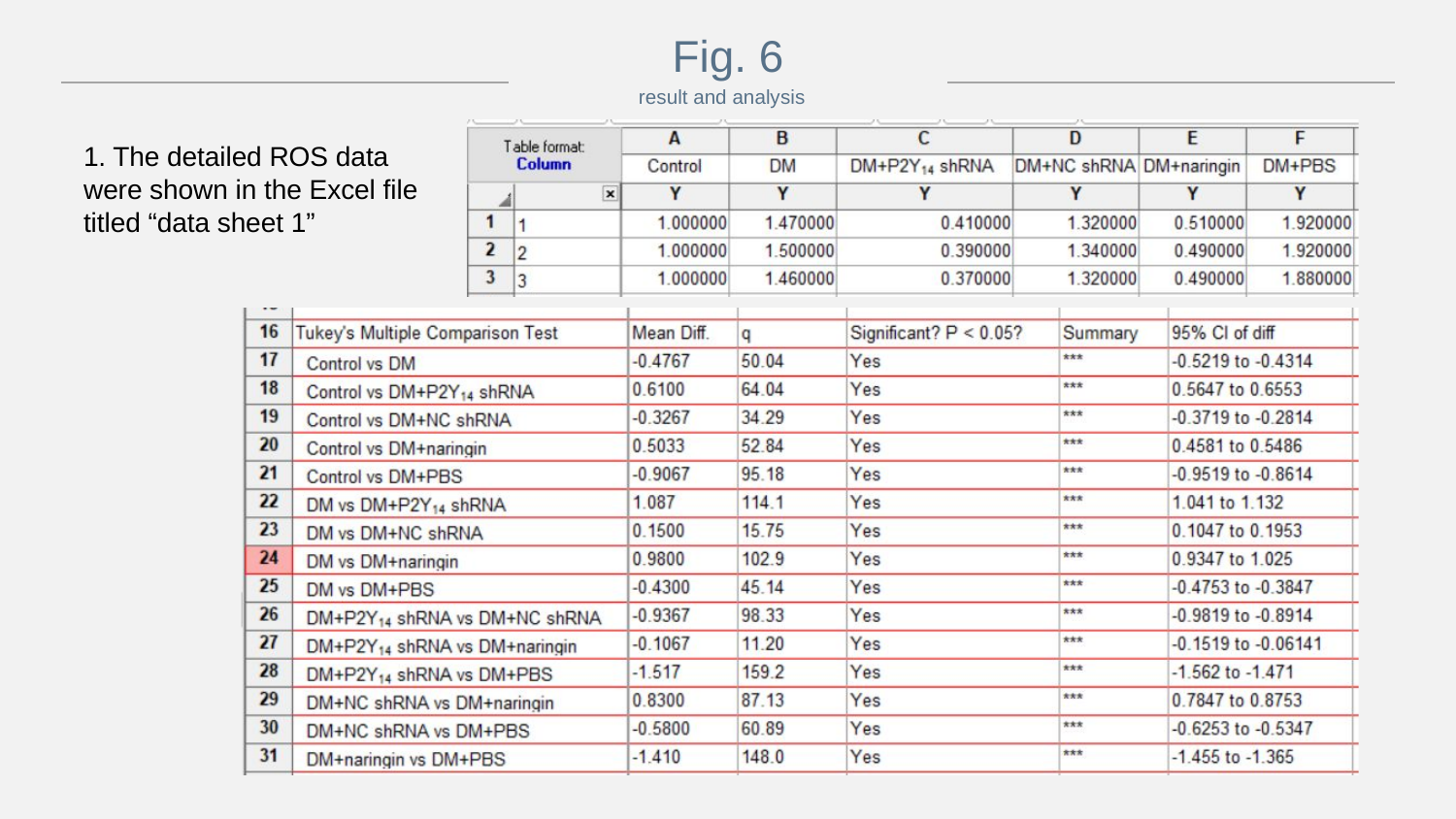

Fig. 6
result and analysis
1. The detailed ROS data were shown in the Excel file titled “data sheet 1”

## Slide 28
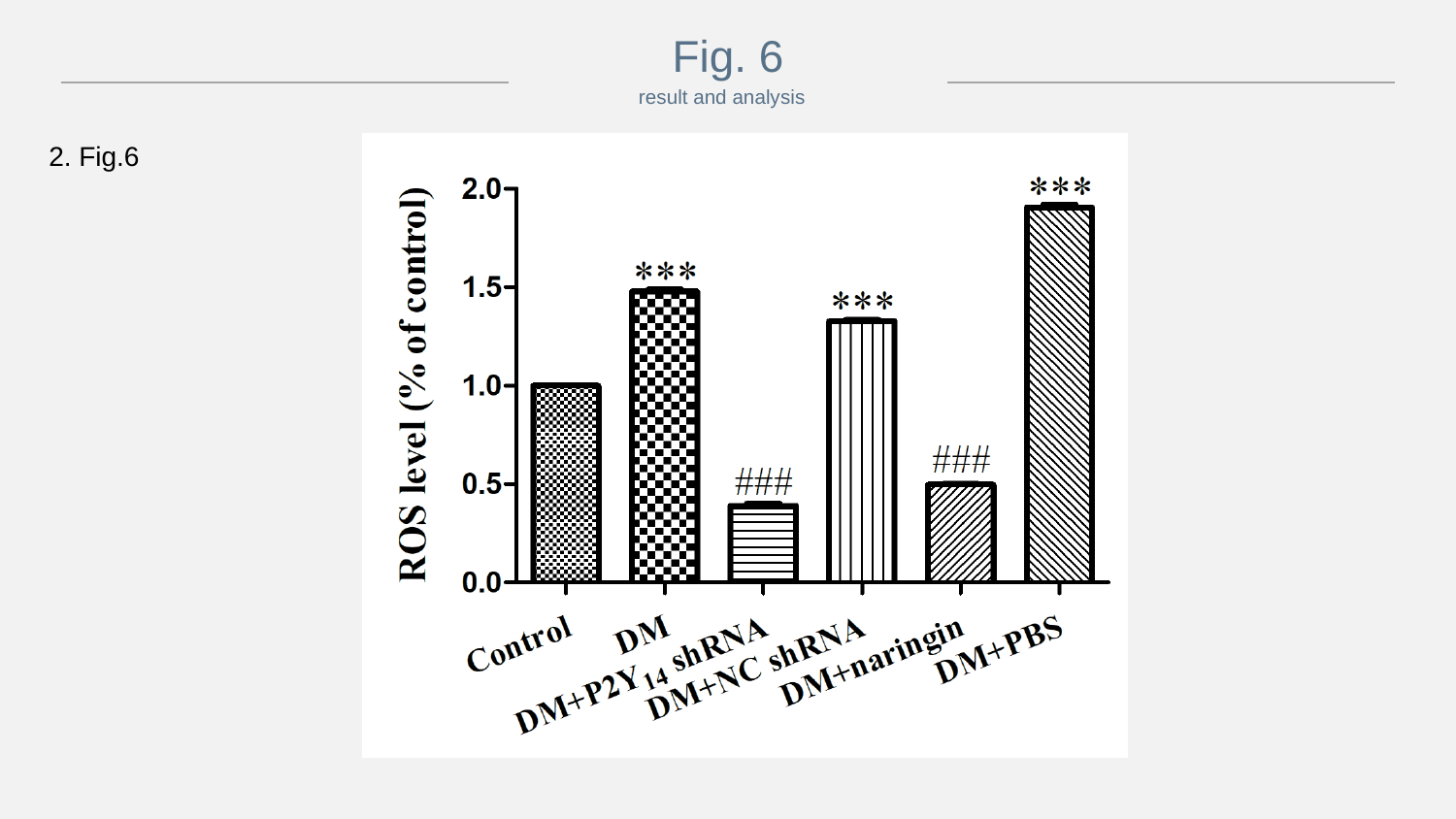

Fig. 6
result and analysis
2. Fig.6

## Slide 29
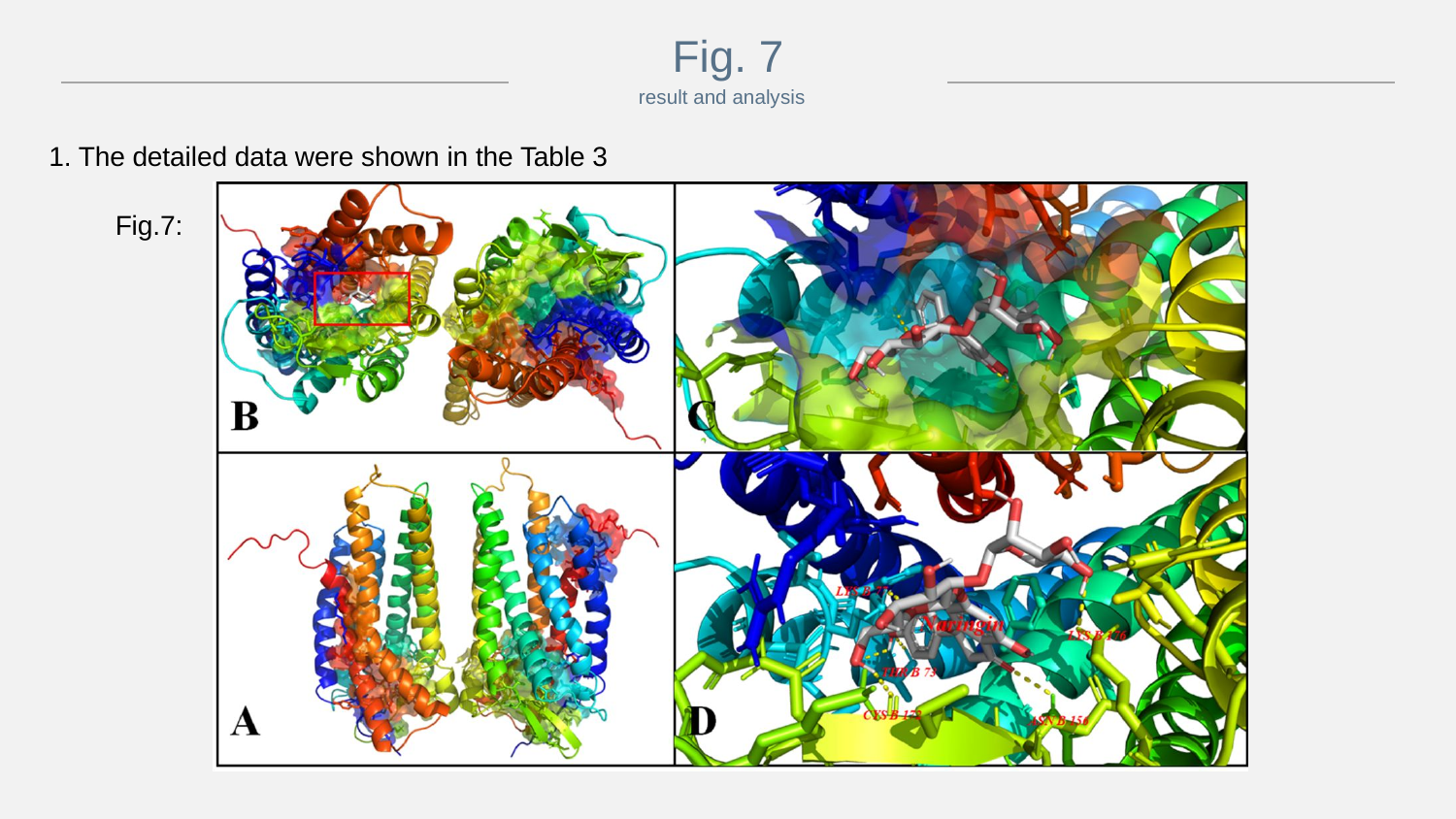

Fig. 7
result and analysis
1. The detailed data were shown in the Table 3
Fig.7:

## Slide 30
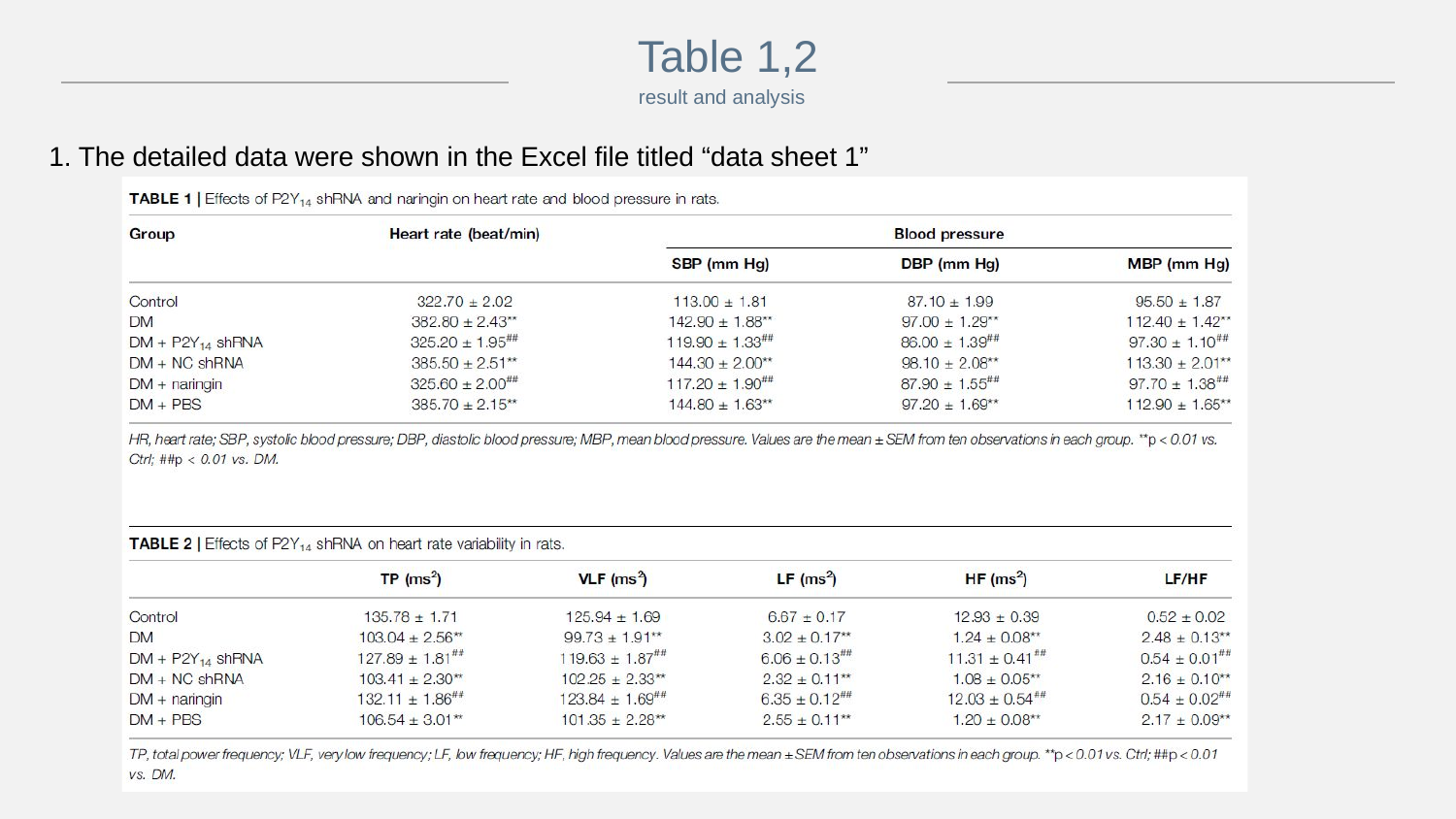

Table 1,2
result and analysis
1. The detailed data were shown in the Excel file titled “data sheet 1”

## Slide 31
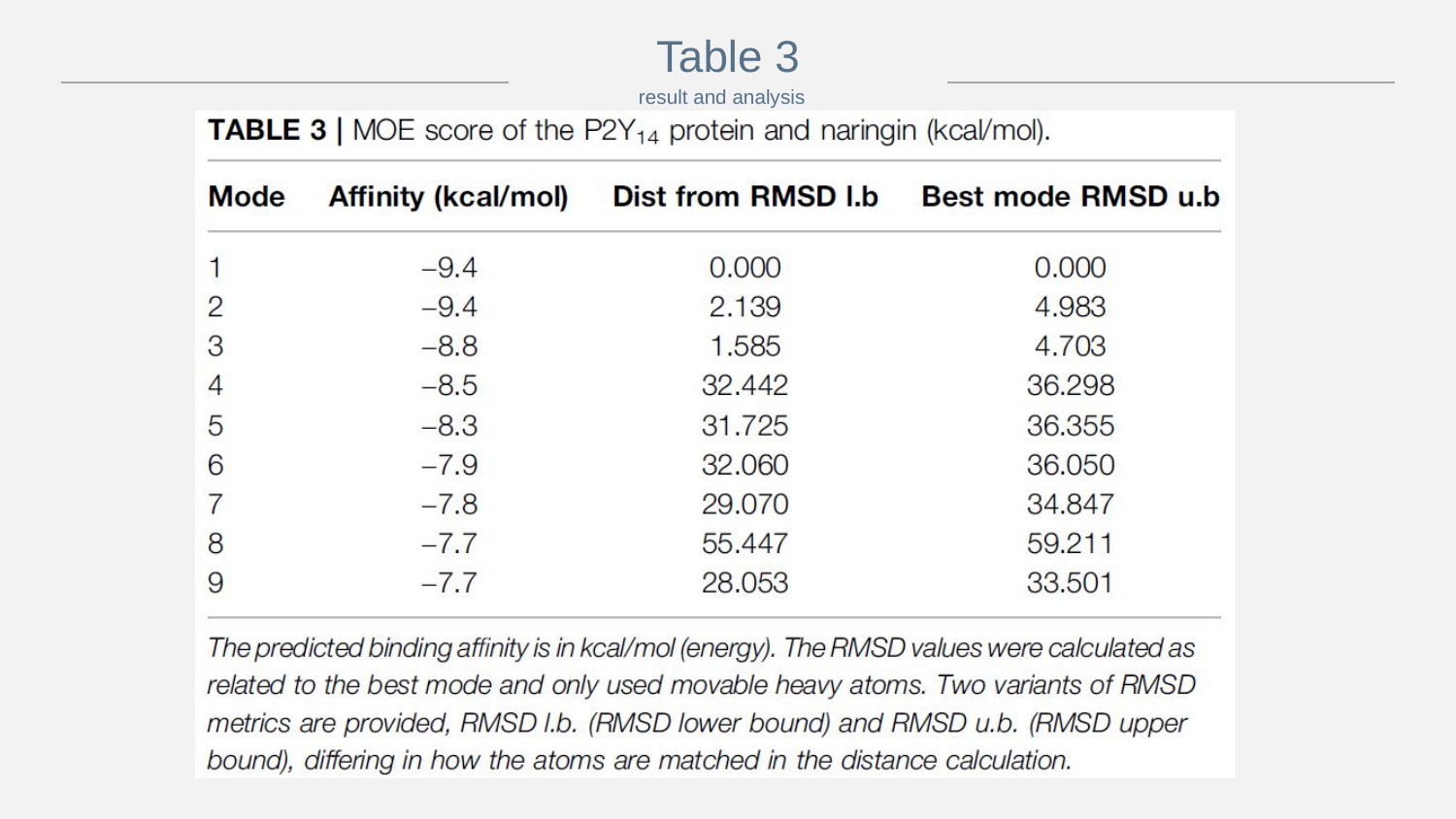

Table 3
result and analysis

## Slide 32
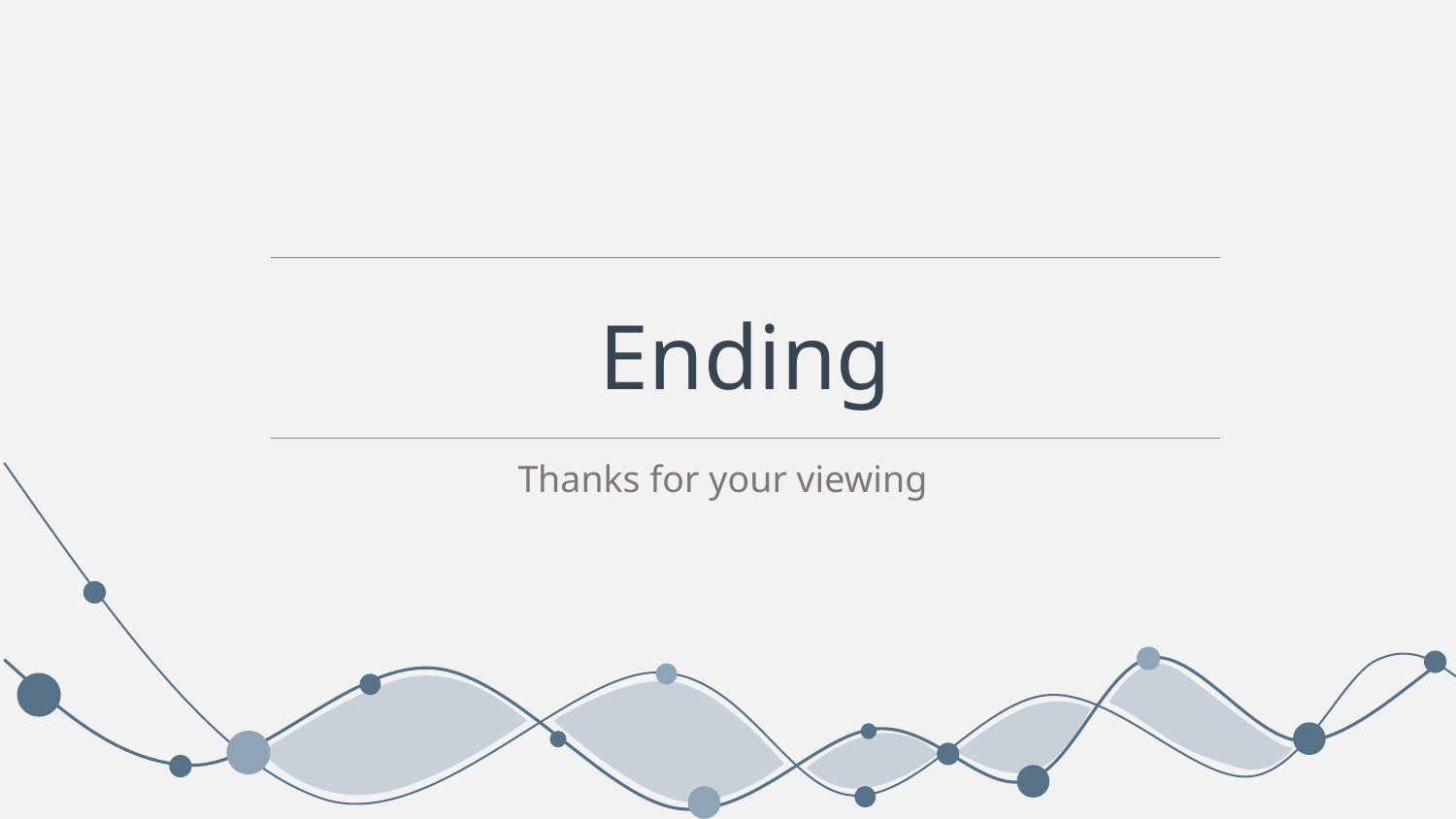

Ending
Thanks for your viewing
